# Reversible formation of coordination bonds in Sn-based metal-organic frameworks for high-performance lithium storage

**DOI:** 10.1038/s41467-021-23335-1

**Published:** 2021-05-25

**Authors:** Jingwei Liu, Daixi Xie, Xiufang Xu, Luozhen Jiang, Rui Si, Wei Shi, Peng Cheng

**Affiliations:** 1 Key Laboratory of Advanced Energy Materials Chemistry (MOE), Renewable Energy Conversion and Storage Center, College of Chemistry, Nankai University, Tianjin, China; 2 Shanghai Synchrotron Radiation Facility, Shanghai Institute of Applied Physics, Chinese Academy of Sciences, Shanghai, China

**Keywords:** Coordination chemistry, Electrochemistry, Batteries, Materials for energy and catalysis

## Abstract

Sn-based compounds with buffer matrixes possessing high theoretical capacity, low working voltage, and alleviation of the volume expansion of Sn are ideal materials for lithium storage. However, it is challenging to confine well-dispersed Sn within a lithium active matrix because low-melting-point Sn tends to agglomerate. Here, we apply a metal-organic framework (MOF) chemistry between Sn-nodes and lithium active ligands to create two Sn-based MOFs comprising Sn_2_(dobdc) and Sn_2_(dobpdc) with extended ligands from H_4_dobdc (2,5-dioxido-1,4-benzenedicarboxylate acid) to H_4_dobpdc (4,4’-dioxidobiphenyl-3,3’-dicarboxylate acid) with molecule-level homodispersion of Sn in organic matrixes for lithium storage. The enhanced utilization of active sites and reaction kinetics are achieved by the isoreticular expansion of the organic linkers. The reversible formation of coordination bonds during lithium storage processes is revealed by X-ray absorption fine structure characterization, providing an in-depth understanding of the lithium storage mechanism in coordination compounds.

## Abstract (web summary)

Tin-based alloying materials have been widely developed for lithium storage, but are limited by large volume variation. Herein, the authors created a new strategy of applying coordination chemistry to construct tin-based metal-organic frameworks which exhibited high-performance lithium storage property.

## Introduction

**Fig. 1:**
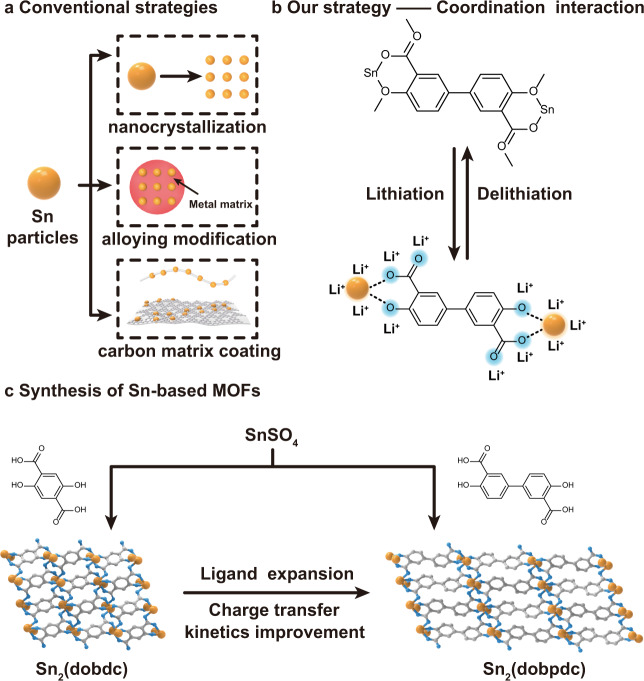
Synthetic strategy for Sn-based MOFs as anode materials. **a** Three conventional strategies for Sn-based anode materials: nanocrystallization, alloying modification and carbon matrix coating. **b** Our strategy uses Sn-based MOFs for reversible lithium storage. **c** Schematic illustration of the synthesis of Sn_2_(dobdc) and Sn_2_(dobpdc).

Rechargeable lithium-ion batteries (LIBs) have been widely applied in portable electronic products to electric vehicles due to their high energy and power densities^1–4^. At present, the most commonly used graphite-based anode materials possess a low theoretical capacity of 372 mAh g^–1^ (based on the formation of LiC_6_) and confront lithium deposition and solvent intercalation issues caused by the fairly low working potential^5,6^. As high-capacity anode materials, Sn-based materials, such as Sn, SnO, and SnO_2_, have been considerably explored because of the natural abundance of Sn, high theoretical capacity of 994 mAh g^–1^ (based on the formation of Li_22_Sn_5_) and suitable anodic voltage^7–11^. However, the large volume variation (~260%) of Sn-based materials during Li alloying and dealloying usually causes the problems of mechanical fracture, swelling of particles and unstable solid-electrolyte interface (SEI) layers, hindering the reaction kinetics and decreasing the cycling stability^12,13^. The strategies of nanocrystallization, alloying modification and application of a carbon matrix coating have been applied to exploit desirable electrode materials to accommodate the volume change and improve the interface contact (Fig. 1a)^8,9,14–19^. However, the relatively low compaction density of the nanomaterials and the large amounts of lithium-inactive matrixes added to the Sn electrodes reduce the energy density of the entire batteries^20^. Moreover, the synthesis of such materials usually requires complex and rigorous processes^21^.

Metal-organic frameworks (MOFs), as a new generation of hybrid and crystalline materials, are constructed with metal nodes and organic linkers through coordination bonds^22–24^ and have promising applications for lithium storage because their regular and tunable compositions and pore structures with tunable surface areas could provide both active sites for lithium storage and well-defined pathways for lithium ion transport^25–30^. The construction of Sn-based MOFs with Sn–O coordination bonds could overcome the drastic volume change of Sn anodes because the coordination interactions can uniformly anchor or release the Sn atoms to the organic matrix and hinder particle growth and aggregation (Fig. 1b). In addition, the organic ligands could also offer active sites for additional lithium storage^31^. By combining the merits of alloy-type metal centers and active organic ligands, well-designed Sn-MOF electrodes could be applied for lithium storage. To the best of our knowledge, the elaborate design and construction of Sn-based MOFs for lithium storage have not been reported. In fact, recent attempts regarding MOFs as anode materials in LIBs mainly involved transition metal-based MOFs with moderate electrochemical performance (Supplementary Table 1), where reversible lithium storage is based on the conversion reaction of metal centers and a charge-state change of the electroactive groups^28–30,32^. Most of them deliver relatively low reversible capacities, especially at high current densities, and are plagued by long-term cycling issues. The lithium storage mechanisms of these reported MOF-based anodes have been investigated by using X-ray photoelectron spectroscopy (XPS), Fourier transform infrared spectroscopy (FTIR), scanning electron microscopy (SEM) and transmission electron microscopy (TEM), which usually provide useful information at the nanoscale, but the atomic accuracy level for coordination compounds is still unclear.

In this paper, we report that high-performance anode materials for LIBs can be obtained by a combination of coordination chemistry and electrochemistry. This approach is demonstrated by two isoreticular Sn-based MOFs from Sn_2_(dobdc) with a small 2,5-dioxido-1,4-benzenedicarboxylate acid (H_4_dobdc) linker to Sn_2_(dobpdc) with an extended 4,4’-dioxidobiphenyl-3,3’-dicarboxylate acid (H_4_dobpdc) linker (Fig. 1c). The coordination units formed by the Sn^2+^ ions and organic ligands can not only effectively utilize the abundant active sites affording high specific capacity but also alleviate the volume change, guaranteeing reversible reactions for long-term cycling stability. Importantly, the extension of the ligand resulted in enhanced utilization of active sites and favorable reaction kinetics for Sn_2_(dobpdc) with an isoreticularly extended framework structure, presenting a high reversible capacity of 1018 mAh g^–1^ over 200 cycles and excellent rate capability, which outperforms other coordination compounds (Supplementary Table 1). Combined studies with in situ powder X-ray diffraction (PXRD), X-ray absorption fine structure (XAFS), high-resolution TEM (HRTEM) characterization, ex situ FTIR and XPS spectroscopy and theoretical modeling provided a detailed analysis of the lithium storage mechanism in these Sn-based MOFs, highlighting that the reversible formation of coordination bonds is the core and foundation to solve particle agglomeration and achieve satisfactory active site utilization in coordination compounds.

## Results

### Structure and basic characterizations

Single-crystal X-ray diffraction studies revealed that both Sn_2_(dobdc) and Sn_2_(dobpdc) crystallize in the monoclinic *P*2_1_/*c* space group (Supplementary Table 2). The framework of Sn_2_(dobpdc) is analogous to Sn_2_(dobdc), featuring the same coordination spheres of Sn^2+^ ions but an enlarged spacing between the Sn^2+^ ions due to the extended dobpdc^4−^ linker. The structure of Sn_2_(dobpdc) is described here representatively. There is one crystallographically independent Sn^2+^ ion that lies in a nonsymmetric disphenoidal coordination environment and is coordinated by four oxygen atoms from three different dobpdc^4-^ ligands (Supplementary Fig. 1a). The Sn–O bond lengths are in the range from 2.065(6) to 2.181(3) Å. The selected bond lengths and angles are provided in Supplementary Table 3, which are in the range of other types of Sn-based coordination compounds^33^. The Sn^2+^ ions are bridged by *μ*_2_-*η*^1^:*η*^1^ carboxylate groups into Sn–COO chains along the *b* axis, which are further connected via dobpdc^4–^ to form a 3D framework (Supplementary Fig. 1b‚c). Topologically, the organic ligand and Sn^2+^ ion can be viewed as six- and three-connected nodes, respectively. Thus, the framework can be viewed as a (3,6)-connected **rtl** net (Supplementary Fig. 2)^34^. Due to the expanded linkers, the distances between the Sn–COO chains increase from 8.8672(12) Å for Sn_2_(dobdc) to 13.1427(16) Å for Sn_2_(dobpdc). The morphologies of Sn_2_(dobdc) and Sn_2_(dobpdc) were observed by SEM (Supplementary Fig. 3), revealing the uniformity of these crystals.

Both Sn_2_(dobdc) and Sn_2_(dobpdc) maintain their phase purity and structural stability after immersion in the electrolyte for 7 days, demonstrating high electrolyte resistance (Supplementary Fig. 4). Thermogravimetric analysis showed that Sn_2_(dobdc) and Sn_2_(dobpdc) are stable up to 500 °C under a nitrogen atmosphere (Supplementary Fig. 5). The thermal stabilities of Sn_2_(dobdc) and Sn_2_(dobpdc) were further evaluated by in situ variable-temperature PXRD measurements on a Pt sample platform in the temperature range from 50 to 600 °C (Supplementary Fig. 6). The PXRD patterns for both MOFs remain unchanged until 500 °C, further demonstrating their excellent thermal stability. The high stabilities of Sn_2_(dobdc) and Sn_2_(dobpdc) are uncommon for Sn-based coordination compounds due to the strong coordination bonds and 3D rigid framework structures, which are suitable for lithium storage studies. Furthermore, the electrical conductivities of Sn_2_(dobdc) and Sn_2_(dobpdc) are 2.9 × 10^−7^ and 9.8 × 10^−7^ S cm^−1^, evaluated by linear sweep voltammetry measurements (Supplementary Figs. 7 and 8).

### Electrochemical performance

**Fig. 2:**
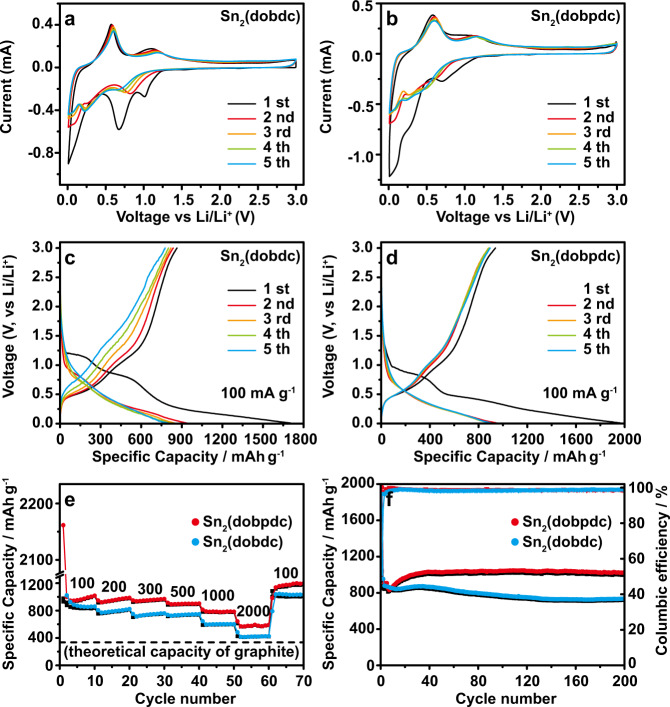
Electrochemical performance of Sn_2_(dobdc) and Sn_2_(dobpdc). **a**, **b** CV curves at 0.1 mV s^–1^ of Sn_2_(dobdc) and Sn_2_(dobpdc). **c**, **d** Discharge–charge profiles of Sn_2_(dobdc) and Sn_2_(dobpdc) in the first five cycles. **e**, **f** Rate performance and cycle stability.

Taking advantage of the multiple lithium storage active sites from the alloying-type Sn centers and electrochemically active groups of organic ligands, Sn_2_(dobdc) and Sn_2_(dobpdc) were evaluated as anode materials for LIBs in CR2032 coin-type cells at 298 K. According to the reversible reaction of Sn-based oxides with lithium^10^, the electrochemical process of Sn-MOFs with lithium is expected to undergo a combination of electrochemical conversion and alloying mechanisms, with the following two steps: Sn_2_L + 4Li^+^ + 4e^–^ ↔ 2Sn + Li_4_L (L^4–^ = dobdc^4−^ or dobpdc^4−^) and Sn + xLi^+^ + xe^–^ ↔ Li_x_Sn (0 ≤ x ≤ 4.4). To investigate this redox chemistry for lithium storage, the cyclic voltammetry (CV) curves for both MOFs were measured at a scan rate of 0.1 mV s^–1^ between 0.01 and 3.0 V (Fig. 2a, b). It is noted that the first cathodic scan with two irreversible cathodic peaks in the ranges of 0.7–1.1 V and 0.3–0.6 V differs from the subsequent scans. This behavior is attributed to the formation of a SEI layer that causes the initial capacity loss^35,36^. The activation of Sn-MOFs to form amorphized materials also occurs during the first lithiation process (a detailed analysis is in the in situ PXRD section). After the first cycle, two pairs of distinctive redox peaks are observed, which are attributed to the gradual insertion or extraction of Li^+^ within the organic ligands and metal centers, respectively. The peak at 0.8 V is due to lithium insertion into the conjugated aromatic ligand with a reduction in the Sn centers, while the peak at 0.2 V is ascribed to the alloying reaction of lithium with Sn. All peaks remain invariable after the first cycle, confirming the presence of reversible and stable electrochemical reactions. Figure 2c, d display the first five galvanostatic discharge–charge profiles of the cells at 100 mA g^–1^. Sn_2_(dobpdc) shows a lower discharge plateau than Sn_2_(dobdc). After the formation of the SEI layers with an initial irreversible capacity loss, both Sn_2_(dobdc) and Sn_2_(dobpdc) exhibit stable lithium storage behaviors, indicating few side reactions of the electrolyte. Two sloping discharge plateaus for Sn_2_(dobdc) are observed in the ranges of 1.2–0.6 V and 0.45–0.15 V, and the discharge plateaus for Sn_2_(dobpdc) are in the ranges of 0.8–0.5 V and 0.4–0.1 V (Fig. 2c, d), which agree with the electrochemical reactions in the CV results. Figure 2e shows the rate performances after cycling at various current densities from 200 to 2000 mA g^–1^. Even at 2000 mA g^–1^, the capacities of the electrodes are still higher than the theoretical capacity of commercial graphite. Figure 2f shows the cycling performance at 200 mA g^–1^. After 200 cycles, the Sn_2_(dobpdc) anode achieves a capacity of 1018 mAh g^–1^, whereas Sn_2_(dobdc) maintains a capacity of 731 mAh g^–1^ at 200 mA g^–1^. The good cycling performances at high currents are also achieved. At 500 mA g^–1^ for 600 cycles, the Sn_2_(dobdc) and Sn_2_(dobpdc) electrodes exhibit stable capacities over 400 and 650 mAh g^–1^, respectively (Supplementary Fig. 9).

Due to the isoreticular structures of Sn_2_(dobdc) and Sn_2_(dobpdc), the distinct lithium storage capacities between Sn_2_(dobdc) and Sn_2_(dobpdc) could be derived from the expansion of the organic linkers. Apart from the alloying reactions of the Sn centers, which are almost the same in these two MOFs, density functional theory (DFT) calculations were performed to evaluate the lithium storage capability of organic ligands. Based on the calculated Li uptake numbers of 8 and 12 for the organic ligands (Supplementary Fig. 10 and Supplementary Tables 4 and 5), Sn_2_(dobdc) and Sn_2_(dobpdc) are able to deliver theoretical capacities of 1044 and 1099 mAh g^–1^, respectively, with contributions from the Sn centers. The practical capacities of the Sn_2_(dobdc) and Sn_2_(dobpdc) are 731 and 1018 mAh g^–1^ at 200 mA g^–1^, corresponding to utilization efficiencies of 70.0% and 92.6%, respectively. The high utilization efficiency of Sn_2_(dobpdc) is attributed to the increased conjugation of the organic ligand, which can realize the full electrochemical activity of both redox active groups and aromatic rings.

**Fig. 3:**
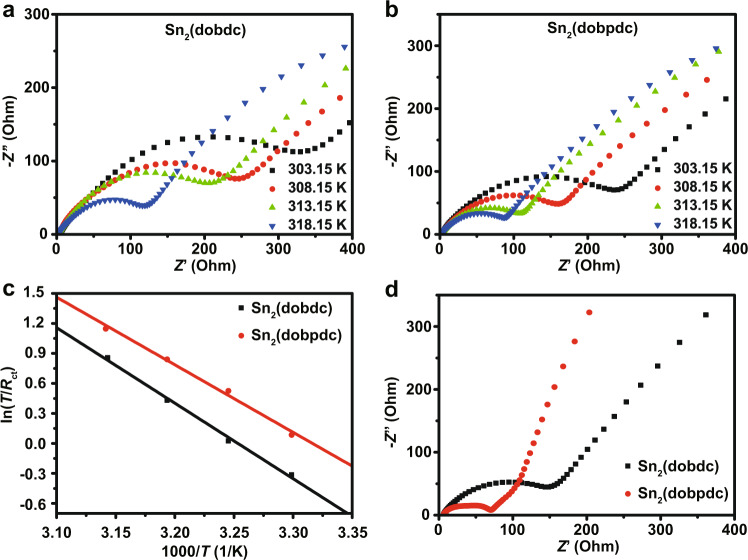
Kinetic analysis of Sn_2_(dobdc) and Sn_2_(dobpdc). **a**, **b** EIS of Sn_2_(dobdc) and Sn_2_(dobpdc). **c** Plots of ln(*T*/*R*_ct_) and 1000/T for Sn_2_(dobdc) and Sn_2_(dobpdc). **d** EIS of the two Sn-MOF electrodes after 200 cycles.

To reveal the difference in the rate performances of Sn_2_(dobdc) and Sn_2_(dobpdc), which mainly depends on the kinetics during the lithium storage processes, electrochemical impedance spectra (EIS) and additional CV characterization were performed. The activation energies of these two Sn-based MOFs for lithium storage were estimated by the EIS data at different temperatures (Fig. 3a–c). In general, a semicircle at high and medium frequencies and a straight line at low frequencies are related to the charge transfer resistance (*R*_ct_) and the Li^+^ diffusion resistance (*R*_w_), respectively. The value of *R*_ct_ decreases as the temperature increases because electrochemical kinetic reactions can easily occur at high temperatures^37^. Sn_2_(dobpdc) exhibits a smaller *R*_ct_ and a lower *R*_w_ than Sn_2_(dobdc), indicating that Sn_2_(dobpdc) undergoes faster reaction kinetics (Fig. 3a, b). Based on the EIS data at different temperatures, the apparent activation energy (*E*_a_) can be calculated by *i*_0_ = *RT*/n*FR*_ct_ and *i*_0_ = *A*exp(−*E*_a_/*RT*)^37,38^, where *i*_0_ is the exchange current, *A* is the temperature-independent coefficient, *R* is the gas constant, *T* is the absolute temperature, n is the number of transferred electrons, and *F* is the Faraday constant. The *E*_a_ value is obtained by the slope of the ln(*T*/*R*_ct_) versus 1000/*T* plot fitted by the Arrhenius equation (Fig. 3c). The calculated *E*_a_ values are 62.68 kJ mol^–1^ and 53.46 kJ mol^–1^ for Sn_2_(dobdc) and Sn_2_(dobpdc), respectively. The relatively low *E*_a_ of Sn_2_(dobpdc) indicates an easier diffusion process for lithium intercalation than that in Sn_2_(dobdc). Figure 3d displays the EIS of these two Sn-MOFs after 200 cycles. The Sn_2_(dobpdc) electrode exhibited a lower *R*_ct_ of 71.8 Ω in comparison to the *R*_ct_ of 175.4 Ω for the Sn_2_(dobdc) electrode, in accordance with the faster reaction kinetics of Sn_2_(dobpdc). Furthermore, the CV tests at different scan rates (0.1–0.4 mV s^–1^) with a voltage range from 0.01 to 3.0 V were also carried out to probe the kinetic origin of the lithium storage in the Sn_2_(dobdc) and Sn_2_(dobpdc) electrodes (Supplementary Fig. 11a, b). The relationship between the log (peak current) and log (scan rate) from the CV curves suggests that a combination of diffusion-controlled and surface-controlled processes contribute to the electrochemical reactions in the Sn_2_(dobdc) and Sn_2_(dobpdc) electrodes (Supplementary Fig. 11c, d)^10^. Quantitative analyses demonstrate that the percentage of capacitive contribution of the Sn_2_(dobpdc) electrode is higher than that of the Sn_2_(dobdc) electrode (Supplementary Fig. 11e, f), confirming that the Sn_2_(dobpdc) electrode has favorable charge transfer kinetics and good rate capability. Furthermore, the diffusion coefficients of Li^+^ in Sn_2_(dobdc) and Sn_2_(dobpdc) were calculated by the galvanostatic intermittent titration technique (GITT) (Supplementary Fig. 12), demonstrating that Sn_2_(dobpdc) has a higher Li^+^ diffusion coefficient than Sn_2_(dobdc). The enhanced kinetics of Sn_2_(dobpdc) are from the high surface area and pore volume of the expanded framework (Supplementary Fig. 13). Therefore, the expanded organic ligand can not only enhance π-aromatic conjugation but also improve the contact area between the electrode and electrolyte, resulting in a high utilization of active sites for a high capacity (Fig. 2f) and fast kinetics for lithium storage (Fig. 2e).

### Lithium storage mechanism

**Fig. 4:**
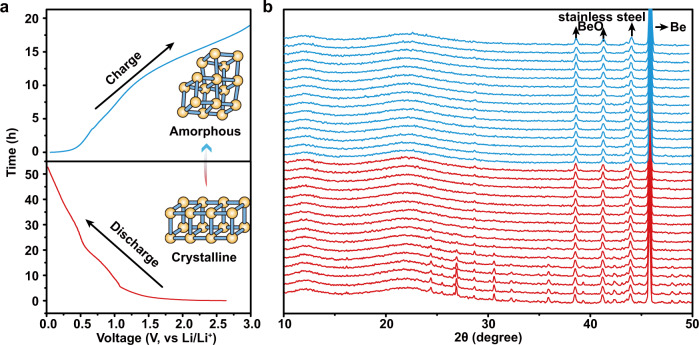
In situ PXRD of the Sn_2_(dobpdc) electrode. **a** Discharge–charge profile of Sn_2_(dobpdc) at 50 mA g^−1^ for the first cycle in the in situ cell. Inset: schematic diagram of the transformation of Sn_2_(dobpdc) from crystalline to amorphous states. **b** In situ PXRD of Sn_2_(dobpdc) collected at different discharge–charge states corresponding to the process in **4a**.

**Fig. 5:**
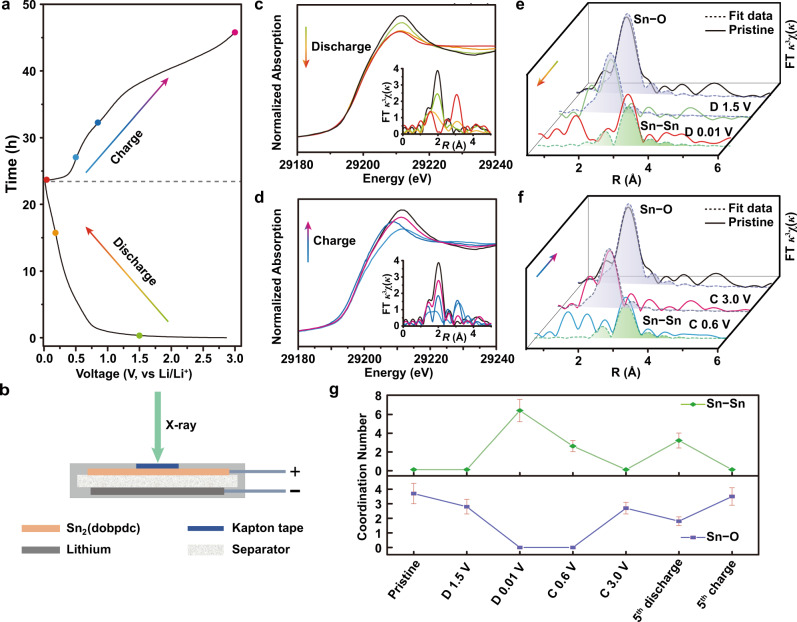
Structural characterization by quasi-in situ XAFS spectroscopy during a lithiation-delithiation cycle of Sn_2_(dobpdc). **a** Load curve during the second discharge–charge process at 50 mA g^−1^ with marked states for quasi-in situ XAFS tests. **b** Schematic cross-section of a coin-type battery for the quasi-in situ XAFS test. **c**, **d** Sn K-edge XANES during the discharge–charge process. The insets display the corresponding Sn K-edge k^3^-weighted FT-EXAFS spectra. **e**, **f** Sn K-edge k^3^-weighted FT-EXAFS spectra fitting in *R* space for selected states during the discharge–charge process. The black lines in **c**-**f** correspond to the XAFS spectra of the pristine electrode. **g** CNs of near neighbors around Sn atoms at different states in the quasi-in situ battery.

**Fig. 6:**
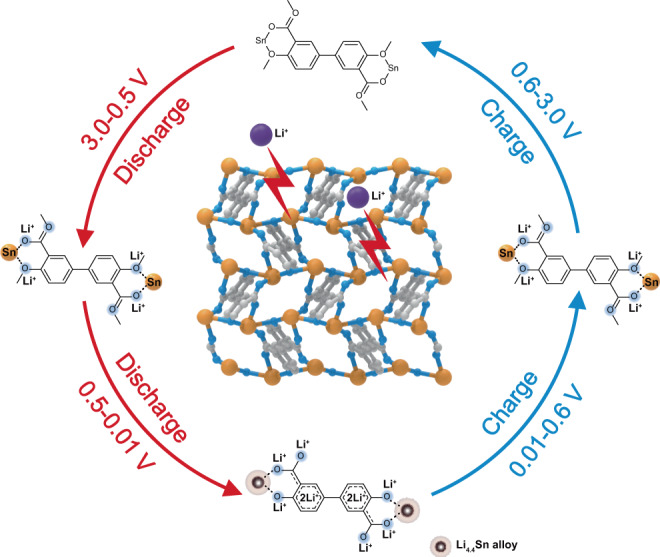
Reaction mechanism of Sn-MOF. Schematic gradual lithiation and delithiation of metal centers and organic ligands of amorphous Sn_2_(dobpdc) during discharge–charge processes.

To determine the lithium storage mechanism of these Sn-based MOFs, in situ and ex situ characterizations were performed to analyze these Sn-MOF-based electrodes during lithiation and delithiation processes (Figs. 4–6, Supplementary Figs. 14–24). Supplementary Fig. 14a and Fig. 4a are the discharge–charge curves for Sn_2_(dobdc) and Sn_2_(dobpdc) at 50 mA g^−1^ during the first cycle, respectively, corresponding to the in situ states for the PXRD measurements, as shown in Supplementary Fig. 14b and Fig. 4b, indicating that the crystallinities of Sn_2_(dobdc) and Sn_2_(dobpdc) decrease during the first lithiation process. No obvious PXRD peaks are observed after the second lithiation and delithiation processes, revealing that both of them lose the long-range ordered structure and become amorphous upon cycling (Supplementary Fig. 15). Ex situ FTIR spectra were measured to further study the formation of the amorphous MOFs during the first cycle (Supplementary Fig. 16). The selected states of the Sn_2_(dobdc) and Sn_2_(dobpdc) electrodes at 100 mA g^–1^ are marked in Supplementary Fig. 16a, d. Taking the case of Sn_2_(dobpdc), when the potential drops to 1.0 V for a specific capacity of approximately 90 mAh g^–1^, the corresponding FTIR spectra at the a and b states are congruent with the initial states. The formation of SEI layer on the electrode surface influences the FTIR analysis after being discharged to 0.7 V in the first cycle (Supplementary Fig. 16f)^39^. Despite the influence of SEI layer, the circled peaks at the fully charged states are similar to those of the pristine electrodes (Supplementary Fig. 16c, f), indicating the structural recovery of the electrode materials. To exclude the influence of SEI layer, Sn 3*d*, O 1*s* and C 1*s* XPS spectra were measured on the electrode materials after argon ion beam sputter etching for 2 min (Supplementary Figs. 17 and 18). The reversible changes of the Sn 3*d*, O 1*s* and C 1*s* XPS peaks indicate the recovery of the coordination structure (Please see detailed discussions in Supplementary Notes after Supplementary Figs. 17 and 18). The amorphization during the first discharge process could endow these active materials with an elastic strain capability to accommodate the volume variation from the alloying reaction of Sn with lithium, which is consistent with the excellent cycling performances of Sn_2_(dobdc) and Sn_2_(dobpdc) (Fig. 2f). In addition, due to the locally remaining coordination structure, these amorphized samples with crystal defects demonstrate a redox active performance that is superior to that of their crystalline counterparts (Fig. 4a). The amorphous Sn-MOFs have similar characters to quasi-MOFs and glassy MOFs in terms of the absence of Bragg peaks in PXRD patterns. The lack of long-range ordered structure could expose more active sites^40–42^.

To study the valence state and local coordination structure evolution around Sn centers in the amorphous materials during cycling, quasi-in situ XAFS spectra were measured corresponding to the marked states, as shown in Fig. 5a, by which X-ray absorption near edge structure (XANES) and extended X-ray absorption fine structure (EXAFS)^43^ could provide fundamental information about the valence state and local coordination structure of the Sn_2_(dobpdc) electrode (Fig. 5 and Supplementary Figs. 19 and 20), respectively. The XAFS spectra of the pristine Sn_2_(dobpdc) electrode confirmed the bivalent state of Sn^2+^ and Sn–O coordination of 2.13 Å with a coordination number (CN) of 3.7±0.7 (Supplementary Fig. 19 and Table 6), which is consistent with the crystal data of Sn_2_(dobpdc) (Supplementary Table 3). The quasi-in situ tests were performed on a treated CR2032 coin battery (Fig. 5b and Supplementary Fig. 21). During the discharge process (Fig. 5c), the amplitude of the white line in the XANES profile of the Sn_2_(dobpdc) electrode slowly decreases as a result of the reduction of Sn centers. During the charge process, the intensity of the main XANES peak of the Sn_2_(dobpdc) electrode gradually increases back to the original state (Fig. 5d). The insets in Fig. 5c, d are the corresponding Fourier transformation-EXAFS (FT-EXAFS) spectra, which could reveal the local coordination structure change around the Sn centers. Upon discharging, the intensity of the Sn–O bonds continuously decreases due to the insertion of lithium into organic ligands and the alloying reaction of lithium with Sn centers. Moreover, a new peak emerges at approximately 3.0 Å because of the formation of Sn–Sn bonds from the alloying reaction. The Sn–O bonds recover, and the Sn–Sn bonds disappear after fully charging, indicating reversible reactions around the Sn centers. To determine the coordination environment around the Sn center, phase-corrected FT-EXAFS of selected states were fitted in *R* space. As shown in Fig. 5e and Supplementary Fig. 20, the Sn–O coordination interactions are broken with a decrease in CN of the Sn–O coordination shell from 3.7 ± 0.7 to 2.8 ± 0.5 after being discharged to 1.5 V, corresponding to partial disengagement of the organic ligands from the Sn centers due to lithiation of the organic ligands. Upon becoming fully discharged, a new peak at 2.92 Å is observed and is ascribed to the Sn–Sn shell from the Li_x_Sn alloy with a CN of 6.3 ± 1.2, confirming the alloying reaction. During the charging process, the CN of the Sn–Sn shell decreases from 6.3 ± 1.2 to 2.5 ± 0.6 after charging to 0.6 V because of the dealloying reaction of Li_x_Sn; after charging to 3.0 V, the Sn–Sn interaction disappears, and the Sn–O shell with a CN of 2.7 ± 0.4 reappears (Fig. 5f) and does not fully return to the original state with a CN of 4 due to the formation of a SEI layer during the initial two cycles (Please see detailed discussions in Supplementary Notes after Supplementary Table 6). XAFS analyses of the lithiated and delithiated electrodes after 5 cycles were conducted to further reveal the reversibility of the lithium storage mechanism (Supplementary Fig. 20). After five cycles, the XANES curve of the Sn_2_(dobpdc) electrode in the fully charged state matches well with that of the pristine state, demonstrating the reversible valence state change of the Sn center (Supplementary Fig. 20a). Based on the FT-EXAFS spectra and fitting results (Supplementary Fig. 20b, c and Fig. 5g), a weak Sn–O shell at 2.15 Å with a CN of 1.8 ± 0.3 can be observed in the discharged electrode after 5 cycles, demonstrating the presence of the Sn–O interaction between the Li_x_Sn alloy and the organic ligands. In the charged electrode after 5 cycles, the peak at 2.15 Å from the Sn–O shell with a CN of 3.5 ± 0.6 is very close to that of the pristine electrode (3.7 ± 0.7), confirming the reconstruction of the coordination environment of Sn. These results demonstrate the reversible transformation between the formation of Sn–O and Sn–Sn bonds during the electrochemical reaction in the Sn_2_(dobpdc) electrode.

To verify the intermediates that form during the alloying reaction around Sn centers, HRTEM analysis of Sn_2_(dobpdc) was performed during the second cycle (Supplementary Fig. 22). Since these MOFs are easily damaged by the electron beam, the lattice fringe information disappears for the pristine states (Supplementary Fig. 23). During the discharge process, the interplanar distances of 0.291, 0.282 and 0.198 nm are consistent with the (200) and (101) lattice planes in Sn and the (933) lattice planes in Li_4.4_Sn, respectively, confirming the formation of Sn and Li_4.4_Sn. These results are consistent with the Li^+^ ions inserted into the active materials with a reduction of Sn(II) to Sn(0) followed by the alloying reaction (Supplementary Fig. 22a). During the charging process, the appearance of the interplanar distance of 0.294 nm for the (200) lattice planes of Sn metal confirms the dealloying reaction of Li_4.4_Sn; after becoming fully charged, the amorphous Sn_2_(dobpdc) recovers with the absence of lattice fringe information (Supplementary Fig. 22b). These HRTEM results confirm the reversible alloying and dealloying of Sn with Li^+^ ions.

Based on the above analyses, the overall lithiation and delithiation reaction processes of Sn_2_(dobpdc) can be described, as shown in Fig. 6. Although the amorphization of Sn_2_(dobpdc) occurs during the first cycle, the coordination interaction between Sn centers and organic ligands could be maintained to sustain stable and long-term lithium storage behavior. In the following processes, the electrochemical reactions in the amorphous Sn_2_(dobpdc) phase are related to two successively reversible processes: the redox reaction of organic ligands and the alloying reaction of Sn centers. During the discharge process, a capacity of ~210 mAh g^–1^ over the voltage range from 3.0 to 0.5 V is related to Li^+^ insertion into organic ligands with a reduction of the Sn centers; after becoming fully discharged, the superlithiation of organic ligands and alloying reaction of Sn could provide a capacity of ~890 mAh g^–1^ for an uptake of approximately 17-Li^+^. During the charge process, the dealloying of Li_4.4_Sn to Sn and the extraction of Li^+^ from organic ligands successively take place, resulting in the reversion of amorphous Sn_2_(dobpdc) for a reversible specific capacity of ~1100 mAh g^–1^. Taking advantage of the multiple lithium storage sites and the reversible formation of the coordination bonds, this Sn-based MOF exhibits a high utilization of active sites and fast kinetics for the merits of high reversible capacity at high rate and excellent cycling stability.

We used ex situ FTIR and SEM to study the composition and morphology changes of the electrodes after long-term cycling (Supplementary Figs. 24 and 25). In the FTIR spectra, after the first, fifth and tenth cycles after becoming fully recharged to 3.0 V, the peaks are not entirely consistent with those of the pristine electrodes because of the lithiation and amorphization of the materials, but the FTIR spectra of the fully charged states after five and ten cycles are consistent with that of the first recharged electrode, indicating the recovery of the coordination units. SEM observations show that the morphologies of the active materials are well retained after long-term cycling (Supplementary Fig. 25), accounting for their stable lithium storage performance during cycling.

## Discussion

In comparison with the lithium storage properties and synthetic methods of reported Sn-based nanomaterials and other typical anode materials (Supplementary Table 7), these two well-designed Sn-based MOFs with uniformly confined Sn centers and organic ligands synthesized through a simple solution method achieve a high reversible capacity and long-term cycling stability, verifying the effectiveness of the coordination chemistry strategy for lithium storage. Notably, the reversible formation of the Sn–O–C bond could commendably restrain pulverization and particle aggregation issues to afford stable cycling performance; moreover, the tin alloying element and lithium active organic ligand provide multiple lithium storage sites, thus ensuring high capacity. The rate performances of the Sn-based MOFs are moderate due to the relatively poor electronic conductivity for coordination compounds, but our results have demonstrated that this issue could be solved by a rational ligand design via the extension of π-conjugated functional groups.

In summary, two Sn-based MOFs were designed, and their application in lithium storage was investigated in detail. The ligand extension of these isoreticular Sn-MOFs resulted in enhanced specific capacities up to 1018 mAh g^–1^ with high rate performance and long-term cycling stability, which had the best lithium storage property among reported coordination compounds. Detailed mechanism analyses for both atomic accuracy and at the nanoscale were performed by a systematic analysis, including XAFS, to reveal a new insight into coordination compounds for lithium storage beyond the previous understanding. The multiple lithium storage sites from the metal centers and organic ligands, the enhanced π-conjugation from the organic ligands, and most importantly, the reversible formation of the coordination bonds during the lithiation and delithiation processes jointly endow the Sn-MOFs with highly reversible capacity, good rate capability and stable cycling performance. The molecular level of the dispersibility of the Sn centers coordinated with organic redox ligands in the framework effectively suppress pulverization and particle aggregation of Sn particles to guarantee capacity stability for long-term cycling performance. Our findings provide new insights into the mechanism of coordination compounds for lithium storage, which benefits the design and construction of high-performance electrode materials.

## Methods

### Preparation of the Sn_2_(dobdc)

NaOH (0.9 mmol, 36 mg) and 2,5-dioxido-1,4-benzenedicarboxylate acid (0.3 mmol, 59.4 mg) were dissolved in 10 mL deionized H_2_O and subsequently poured into a 10 mL aqueous solution of SnSO_4_ (1.2 mmol, 257 mg). The mixture was heated to 353 K for 72 h. Dark yellow crystals were obtained by filtration, washed several times with water and ethanol, and then fully desolvated by heating under dynamic vacuum at 523 K for 24 h (yields 66% based on SnSO_4_). Elemental analysis for Sn_2_C_8_O_6_H_2_ yielded a calcd (%) for C of 22.27 and H of 0.47; the found (%) for C was 22.24, and for H, it was 0.52.

### Preparation of the Sn_2_(dobpdc)

Similar to the synthetic procedure of Sn_2_(dobdc), 2,5-dioxido-1,4-benzenedicarboxylate acid was replaced with 4,4’-dioxidobiphenyl-3,3’-dicarboxylate acid. Elemental analysis for Sn_2_C_14_O_6_H_6_ yielded a calcd (%) for C of 33.13 and H of 1.19; the found (%) for C was 33.54, and for H, it was 1.45.

### Characterization

The PXRD measurements were performed on a Rigaku Smartlab SE instrument. Elemental analysis was conducted with a Vario EL cube elemental analyzer. Thermal gravimetric analysis was conducted under a nitrogen atmosphere using a METTER TOLEDO TGA 2-Thermogravimetric Analyzer. The FTIR spectra were acquired using a Bruker Alpha ATR-FTIR instrument. The morphologies were observed by field-emission SEM (JEOL, JSM7500F) and HRTEM (Philips Tecnai FEI, 200 kV). XPS spectra were conducted on a Kratos AXIS Ultra DLD spectrometer (UK) with an Al Kα X-ray source.

### Electrochemical measurements

Electrochemical measurements were conducted in CR2032 coin cells with Sn-MOFs as the active material in the working electrode, metallic lithium foil as the counter electrode, Celgard 2300 as the separator and 1 M LiPF_6_ in ethylene carbonate (EC) and diethylcarbonate (DEC) (1:1 v/v) as the electrolyte. The dosage of electrolyte in each cell is 30 μL. All the assembly processes for the coin cells were completed in an Ar-filled glovebox. To prepare the working electrode, 70 wt% Sn-MOF, 20 wt% Ketjen black (KB) and 10 wt% polyvinylidene fluoride (PVDF) were mixed using N-methyl-2-pyrrolidone (NMP) as the solvent. Then, the slurries were coated on Cu foils and dried at 80 °C overnight in a vacuum. The thickness of the electrode is about 15 μm on the Cu foil (Supplementary Fig. 26). The loading amount of Sn-MOF is 1.3–1.5 mg cm^–2^. Galvanostatic discharge–charge experiments were measured on a LANHE-CT2001A test system (Wuhan, China) at room temperature over a voltage range from 0.01 to 3.0 V at different current densities. The CV and EIS were conducted using an electrochemical workstation (CHI 660E). The GITT was conducted at 30 mA g^–1^ for 20 min followed by a rest time of 1 h.

### Quasi-in situ XAFS measurements

The XAFS spectra at the Sn K (*E*_0_ = 29200 eV) edge were obtained at the BL14W1 beamline in the Shanghai Synchrotron Radiation Facility (SSRF) that was operated at 3.5 GeV under “top-up” mode with a constant current of 240 mA. The home-made open-hole batteries with ~2 mg cm^–2^ Sn-MOFs were treated to specific voltage states on LAND-CT2001A battery-testing instruments and then moved to the analysis chamber for further XAFS analysis. The XAFS data were recorded under fluorescence mode with a Lytle ion chamber. The energy was calibrated according to the absorption edge of a pure Sn foil. Athena and Artemis codes were used to extract the data and fit the profiles. For the XANES, the experimental absorption coefficients as a function of the energy *μ*(*E*) were processed by background subtraction and normalization procedures and reported as “normalized absorption” with *E*_0_ = 29200 eV for all the measured samples and a Sn foil/SnO/SnO_2_ standard. For the EXAFS, the data that underwent FT in *R* space were analyzed by applying the first-shell approximate and metallic Sn model for Sn-O and Sn-Sn contributions. The passive electron factors, *S*_0_^2^, were determined by fitting the experimental data on Sn foils and fixing the CN of Sn-Sn to be the theoretical value from the structural model, and then fixed for further analysis of the measured samples. The parameters describing the electronic properties (e.g., correction to the photoelectron energy origin, *E*_0_) and local structure environment, including the *CN*, bond distance (*R*) and Debye-Waller factor around the absorbing atoms, were allowed to vary during the fitting process. The fitted ranges for *k* and *R* spaces were selected to be *k* = 3−11 Å^−1^ with *R* = 1.0−3.5 Å (*k*^3^ weighted).

### DFT calculations

Geometry optimization and frequency analysis of the species were performed by the B3LYP functional^44^ and 6-311 + G(d,p) basis set. All calculations were performed with Gaussian 16^45^. Computed structures were illustrated using CYLView^46^.

### Single-crystal X-ray structure determination

Single-crystal X-ray diffraction data were obtained at 120 K via an Oxford Cryo stream system on a Supernova X-ray diffractometer with graphite monochromatic Mo Kα radiation (*λ* = 71.073 pm). The structures were solved with direct methods and refined by least-squares methods on *F*^*2*^ using SHELXS and SHELXL, respectively, in the Olex2 software packages^47^. All hydrogen atoms were located and refined isotropically^48,49^. The crystallographic data are provided in Supplementary Tables 2 and 3.

## Supplementary information

Supplementary Information

## Data Availability

All the data of this study are available. The authors declare that the data supporting the findings of this study are available within the article and its Supplementary Information files. The X-ray crystallographic data for Sn_2_(dobdc) and Sn_2_(dobpdc) have been deposited at the Cambridge Crystallographic Data Centre (CCDC), under deposition numbers 2007076 and 2007077, respectively. These data can be obtained free of charge from the CCDC via www.ccdc.cam.ac.uk/data_request/cif. The data that support the findings of this study are available from the corresponding author.
