# Supplementary material for: Reversible formation of coordination bonds in Sn-based metal-organic frameworks for high-performance lithium storage
Source: Nat Commun. 2021 May 25;12:3131. doi: 10.1038/s41467-021-23335-1 (PMC8149848; doi:10.1038/s41467-021-23335-1)
Supplement: Supplementary file 1 — Supplementary Information [file 41467_2021_23335_MOESM1_ESM.pdf]

Supplementary information for

Reversible formation of coordination bonds in Sn-based metal-organic frameworks for high-performance lithium storage

Liu et al.

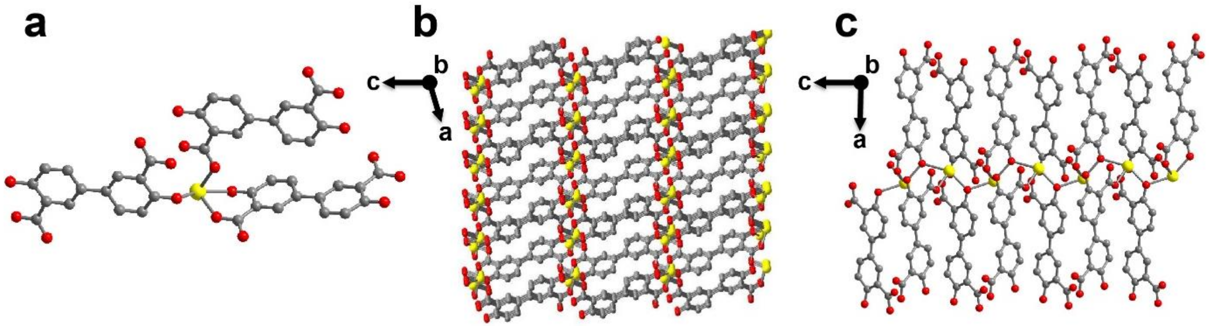

Supplementary Figure 1. (a) Coordination environment of Sn^{2+} ion in Sn_2(dobpdc). (b) 3D structure of Sn_2(dobpdc) along the *b* axis. (c) The 1D chain linked by hydroxyl groups along the *c* axis. The yellow, gray and red balls represent Sn, C and O atoms, respectively.

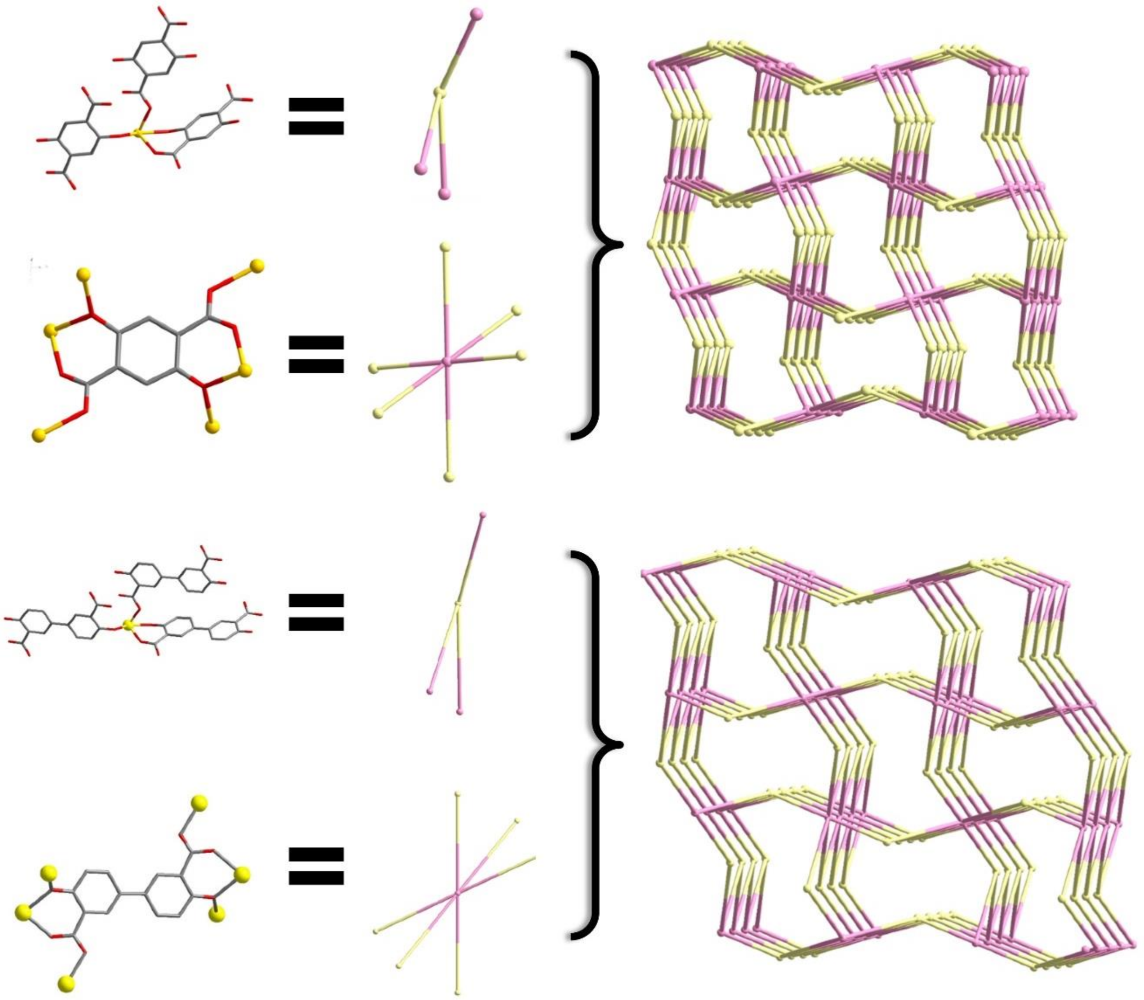

Supplementary Figure 2. Simplified topological structure of Sn_2(dobdc) and Sn_2(dobpdc). The framework can be viewed as a (3,6)-connected **rtl** net with the short (Schläfli) vertex symbol of $\{4\cdot6^{\wedge2}\}_2\{4^{\wedge2}\cdot6^{\wedge10}\cdot8^{\wedge3}\}$.

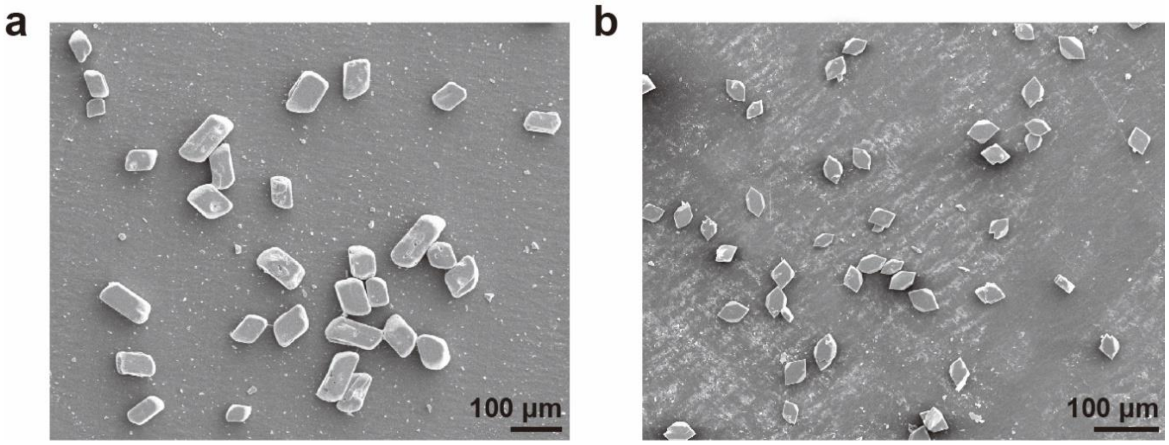

Supplementary Figure 3. SEM images of Sn_2(dobdc) (a) and Sn_2(dobpdc) (b) crystals.

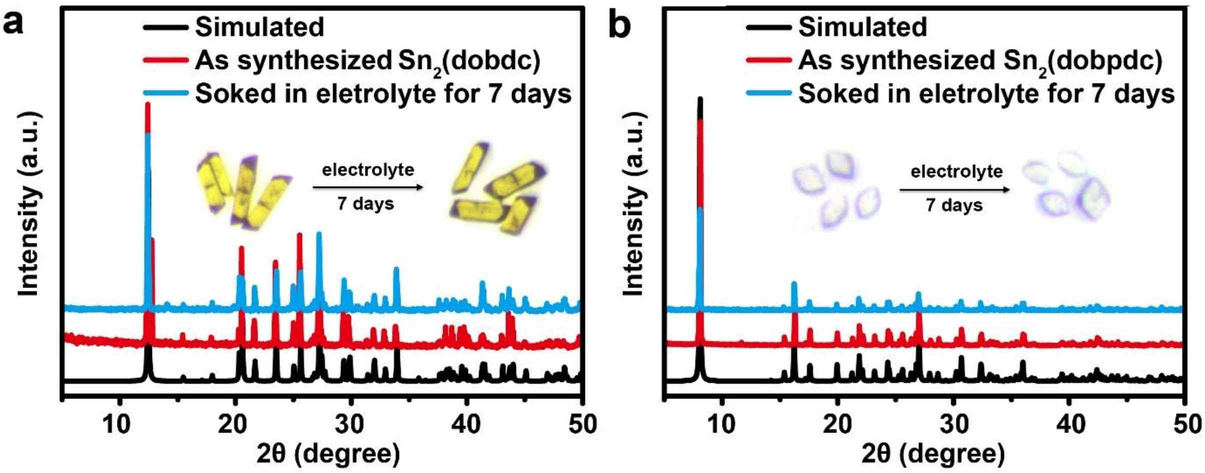

Supplementary Figure 4. PXRD patterns. Simulated and experimental PXRD patterns of Sn_2(dobdc) (a) and Sn_2(dobpdc) (b). Insert: the optical photographs of the crystals for electrolyte stability tests.

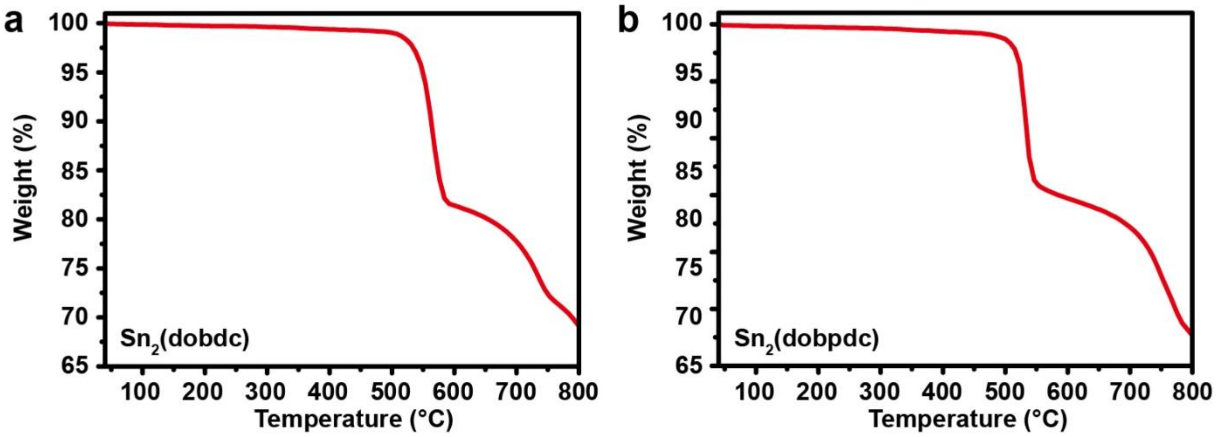

Supplementary Figure 5. TGA patterns. TGA curves of Sn_2(dobdc) (a) and Sn_2(dobpdc) (b).

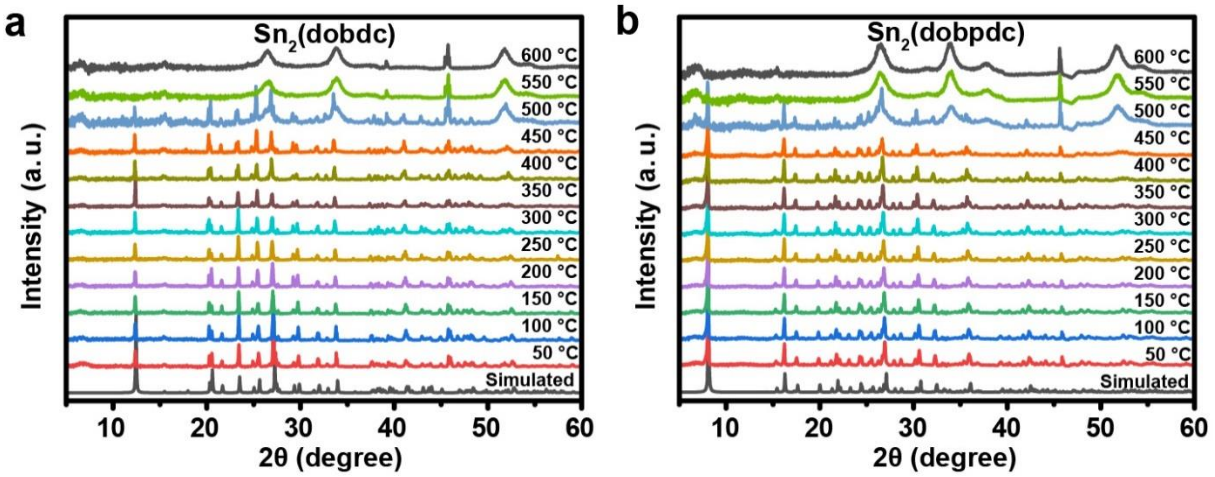

Supplementary Figure 6. PXRD patterns. The in situ variable-temperature PXRD patterns for Sn_2(dobdc) (a) and Sn_2(dobpdc) (b) measured on a Pt sample platform under N_2 atmosphere in the temperature ranges of 50-600 °C. The peaks observed at ca. 46.3 degree is from the Pt sample platform.

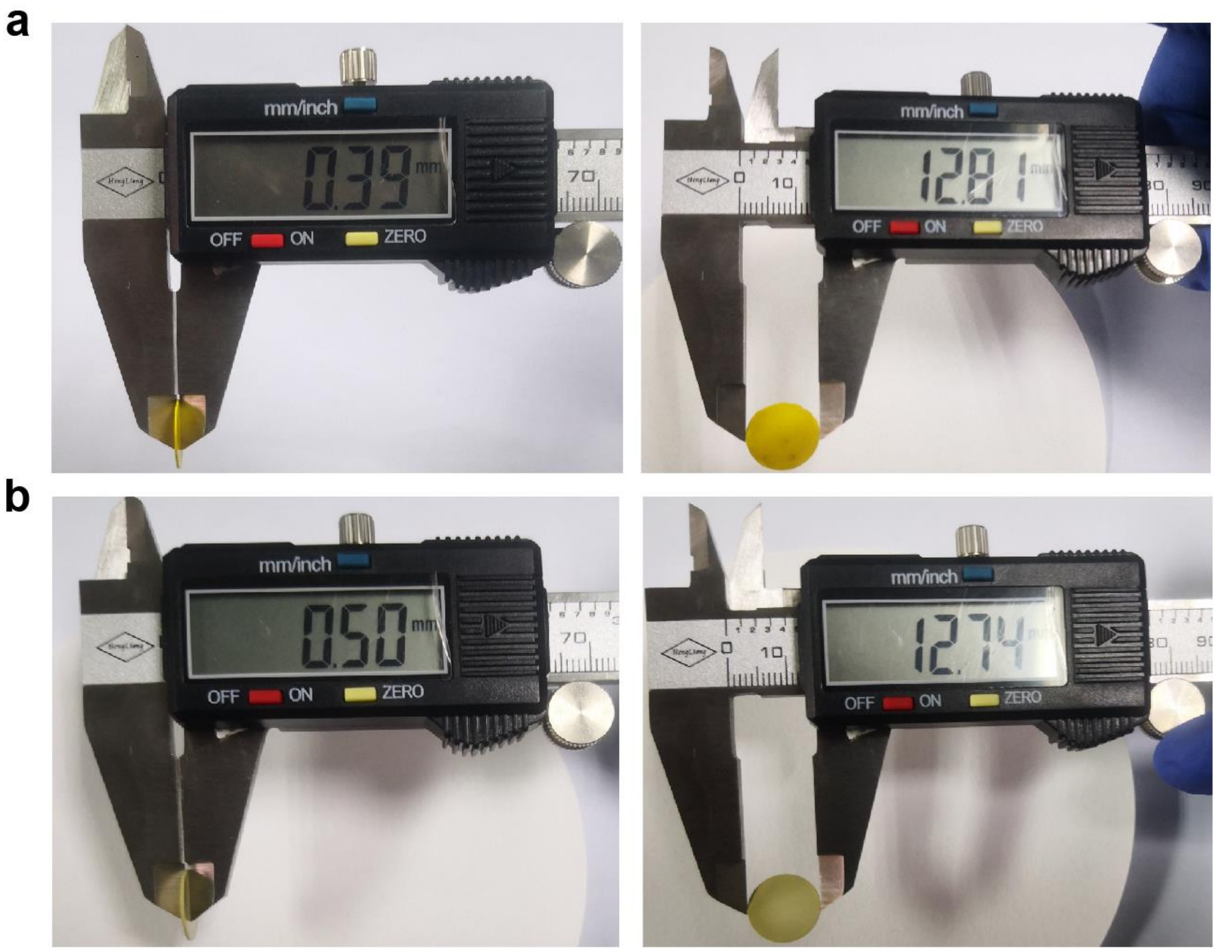

Supplementary Figure 7. The pellets of Sn_2(dobdc) (a) and Sn_2(dobpdc) (b) for electrical conductivity test.

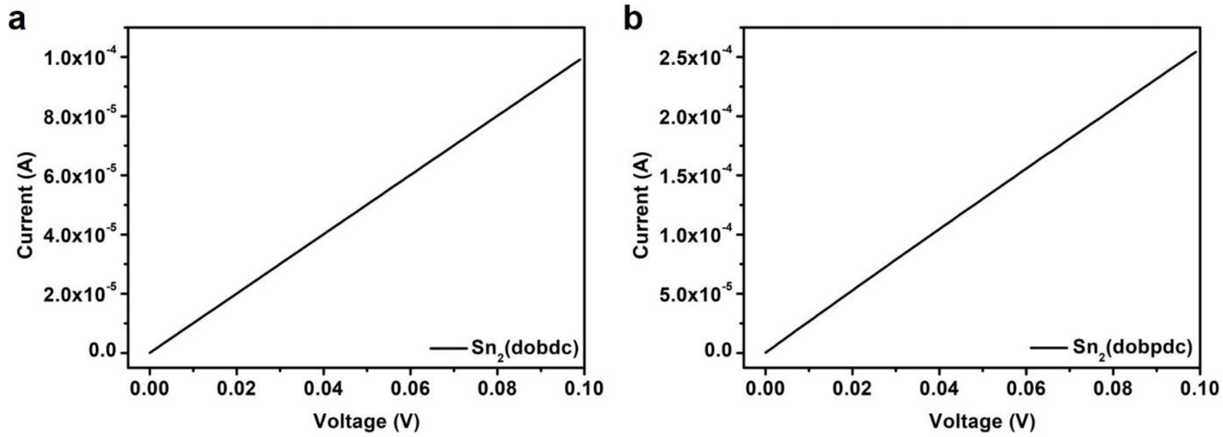

Supplementary Figure 8. Linear sweep voltammetry curves of Sn_2(dobdc) (a) and Sn_2(dobpdc) (b).

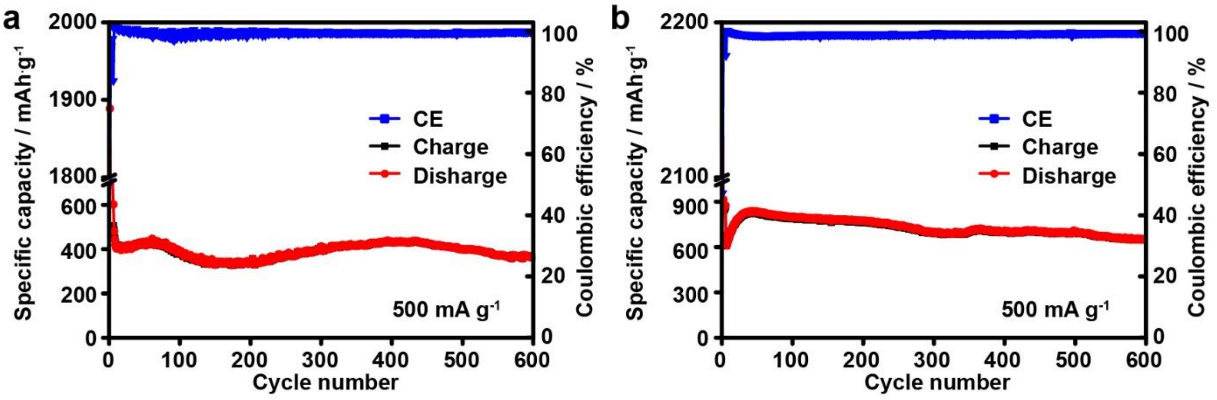

Supplementary Figure 9. Cycling performances of Sn_2(dobdc) (a) and Sn_2(dobpdc) (b) electrodes at 500 mA g^{-1}. The reversible capacities of Sn_2(dobdc) and Sn_2(dobpdc) electrodes can maintain at 400 and 650 mAh g^{-1} after 600 cycles, respectively.

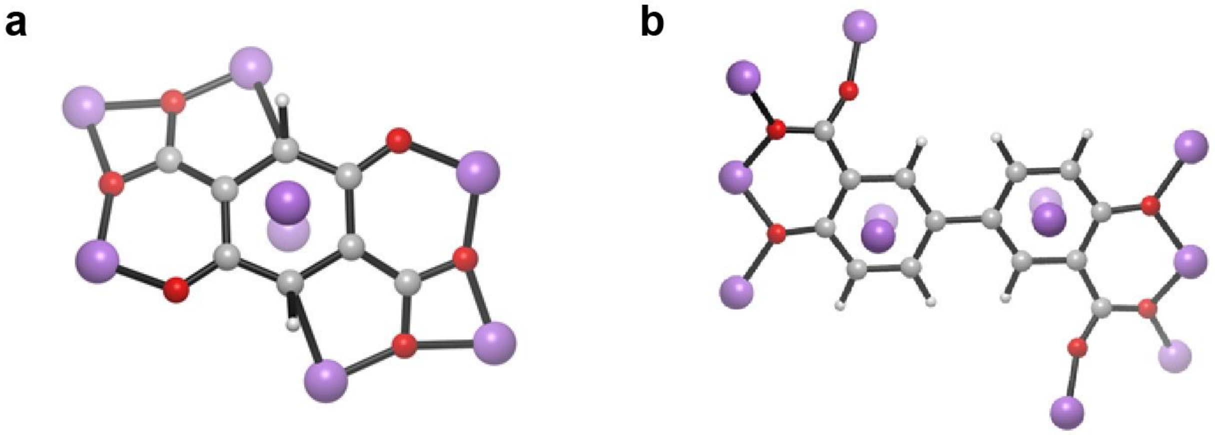

Supplementary Figure 10. Calculated lithium storage sites of the organic ligands of Sn_2(dobdc) (a) and Sn_2(dobpdc) (b).

Theoretical capacity calculation: take the case of Sn_2(dobdc), the structure unit of Sn_2(dobdc) can reversibly alloy 8.8 Li^+ ions with 2.0 Sn and insert 8.0 Li^+ ions with one organic ligand including the insertion to functional groups and the superlithiation of the aromatic ligand. Theoretical capacity is calculated by the equation: $C_{theo} = nF/3.6M$, where n is the number of electrons transferred, F is the Faraday constant, M is the relative molecular mass (g mol^{-1}).

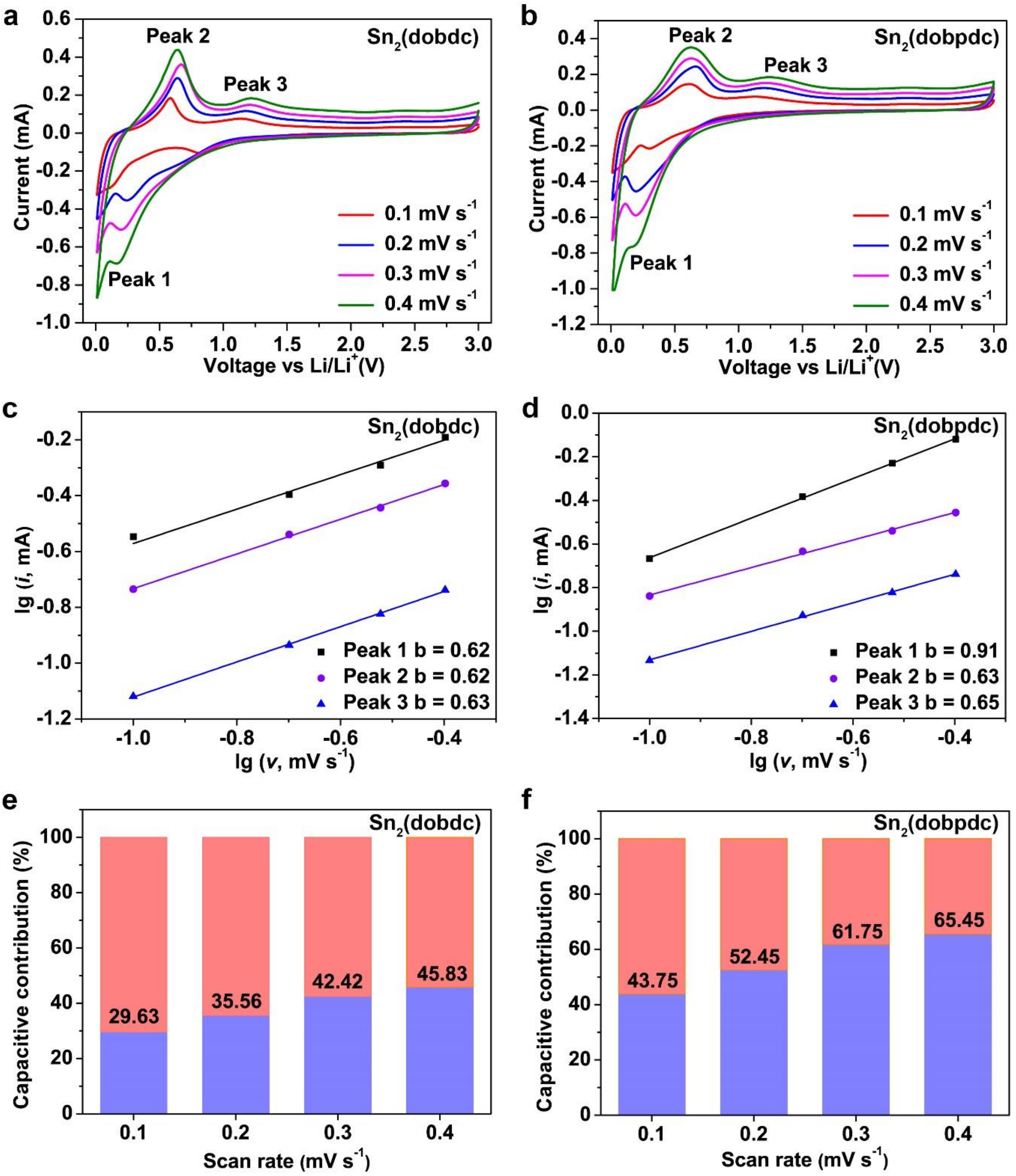

Supplementary Figure 11. Kinetics and quantitative analysis of Li^+ storage mechanism. CV curves of Sn_2(dobdc) (a) and Sn_2(dobpdc) (b) electrodes at different scan rates. The corresponding plots of log (peak current) vs. log (scan rate) at each peak of Sn_2(dobdc) (c) and Sn_2(dobpdc) (d). The capacitive contributions of Sn_2(dobdc) (e) and Sn_2(dobpdc) (f) at different scan rates.

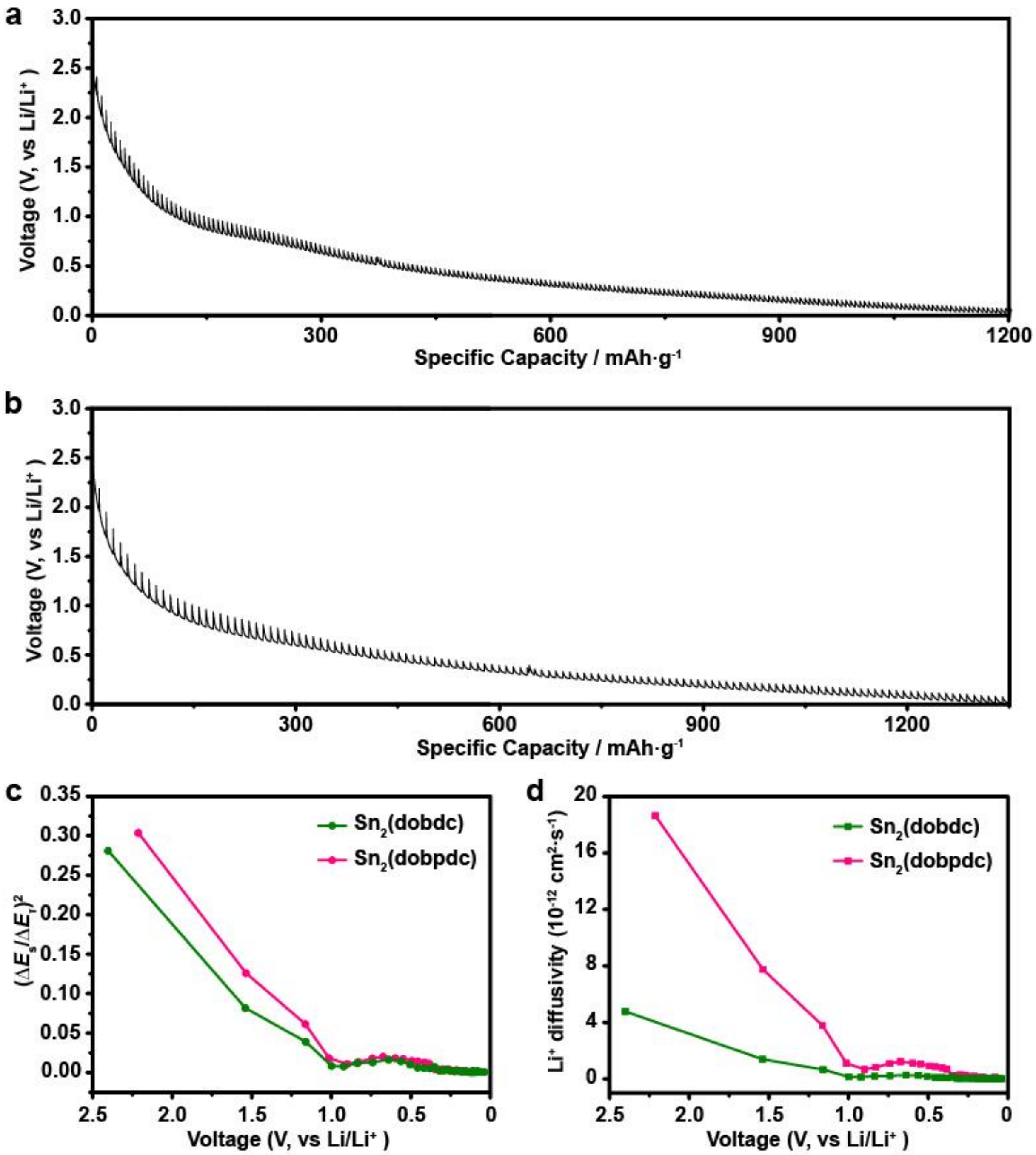

Supplementary Figure 12. The GITT results. (a, b) GITT curves for Sn_2(dobdc) and Sn_2(dobpdc). (c) The values of $(\Delta E_s/\Delta E_\tau)^2$. (d) Diffusion coefficients calculated from GITT versus state of discharge of Sn_2(dobdc) and Sn_2(dobpdc).

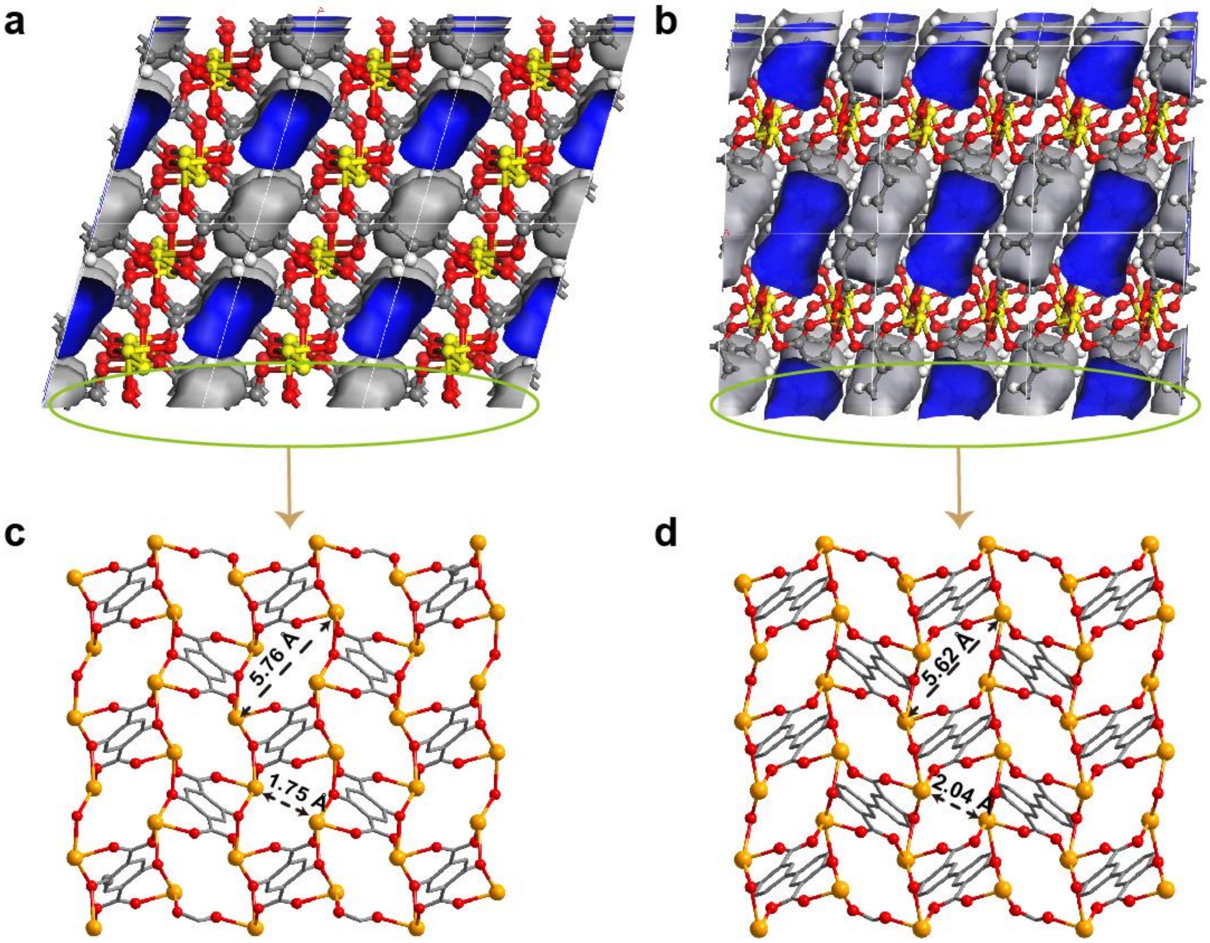

Supplementary Figure 13. Schematic diagrams of the pore surfaces of Sn_2(dobdc) (a) and Sn_2(dobpdc) (b) calculated by Materials Studio. Bottom view of the pores of Sn_2(dobdc) (c) and Sn_2(dobpdc) (d). It is noteworthy that the void space (13.6%) for Sn_2(dobpdc) is significantly larger than that (5.2%) of Sn_2(dobdc), which is attributed to the expanded organic linker. Thus, the calculated surface area (89.75 m^2 g^{-1}) of Sn_2(dobpdc) is higher than that (51.56 m^2 g^{-1}) of Sn_2(dobdc), which is beneficial for facilitating mass/charge transport and interface interaction. For these two Sn-MOFs, the shortest distances of the slits are less than 2 Å, which are both inaccessible for the gases including N_2, CO_2, Ar for the experimental surface area measurement.

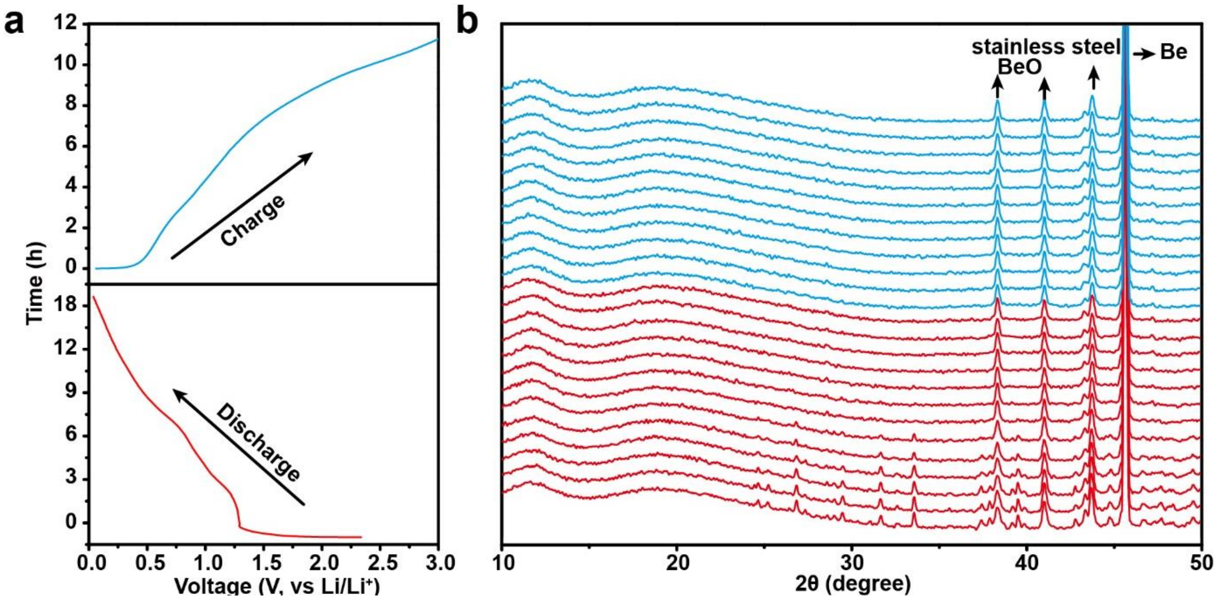

Supplementary Figure 14. In situ PXRD and ex situ FTIR analysis of Sn_2(dobdc) electrode. (a) Discharge–charge profile of Sn_2(dobdc) at 50 mA g^{-1} in the first cycle. (b) In situ PXRD of Sn_2(dobdc) collected at different discharge–charge states corresponding to the process in (a).

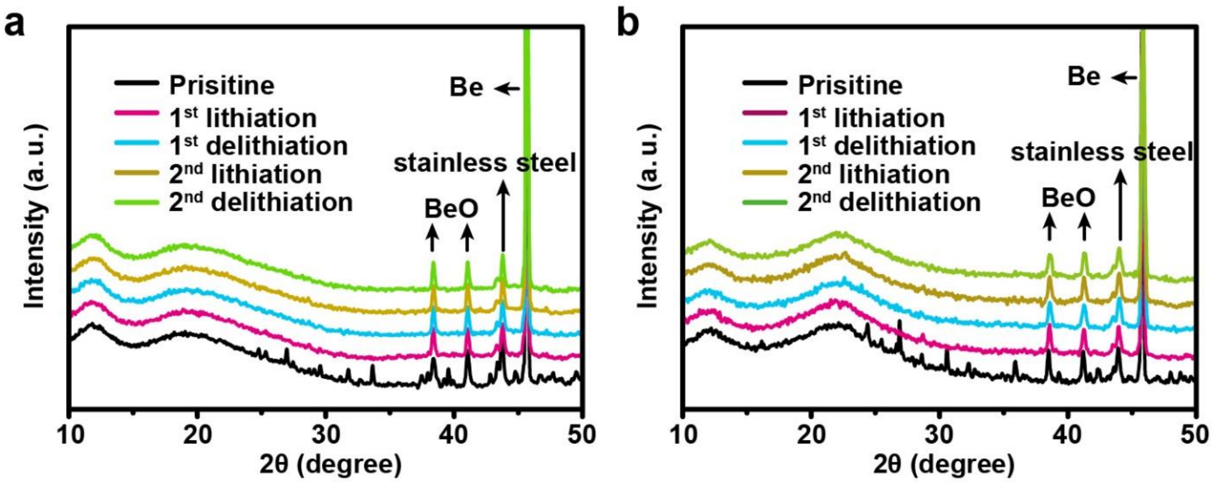

Supplementary Figure 15. Selected in situ PXRD patterns at the different states of Sn_2(dobdc) (a) and Sn_2(dobpdc) (b) electrodes during the first two cycles.

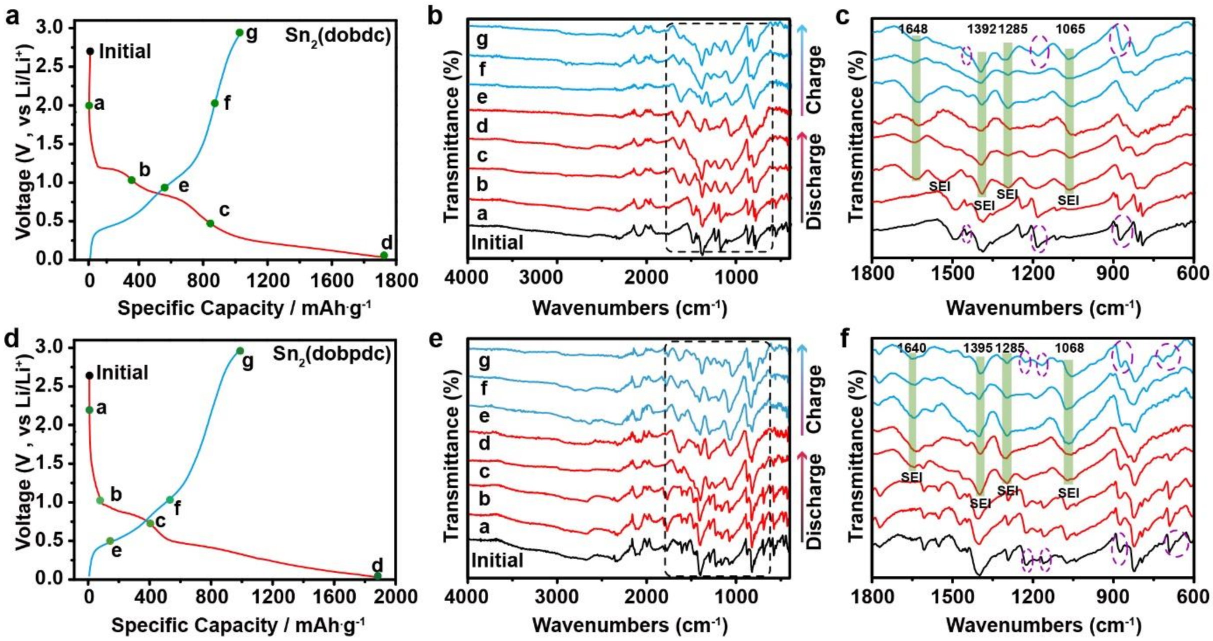

Supplementary Figure 16. Discharge–charge profiles of Sn_2(dobdc) (a) and Sn_2(dobpdc) (d) at 100 mA g^{-1} in the first cycle. Ex situ FTIR spectra of Sn_2(dobdc) (b) and Sn_2(dobpdc) (e) at selected states, as shown in a, d. Magnification of FTIR spectra of Sn_2(dobdc) (c) and Sn_2(dobpdc) (f) in the range from 600 to 1800 cm^{-1}.

For the fresh Sn_2(dobdc) electrode, the FTIR peaks at 1495, 1355, and 1184 cm^{-1} are assigned to the asymmetric stretching vibration of COO^-, the symmetric stretching vibration of COO^- and the stretching of C–O, respectively, under the coordination effects of Sn ions[S1,S2].

After being discharged to 0.5 V (state c), the FTIR peaks at 1640, 1390, 1285, and 1065 cm^{-1} are related to the asymmetric stretching vibration of COO^-, the bending vibration of CH_2, the symmetric stretching vibration of COO^- and the stretching of C–O bonds, which is attributed to the lithium alkyl carbonate $(CH_2OCO_2Li)_2$ of SEI layer[S3,S4].

It is difficult to observe the FTIR peaks of Sn-MOF electrode after the formation of SEI layer around the nanoparticles of Sn-MOF surfaces. As shown in Supplementary Figures 16c and f, the circled peaks at the fully charged states do not belong to the composition of SEI layer, and these peaks are very similar to those of the fresh electrodes. Typically, the circle peaks at 1184 and 1160 cm^{-1} can be attributed to the stretching of C–O in Sn_2(dobdc) and Sn_2(dobpdc) electrodes, respectively. These results imply the structural recovery of the electrode materials.

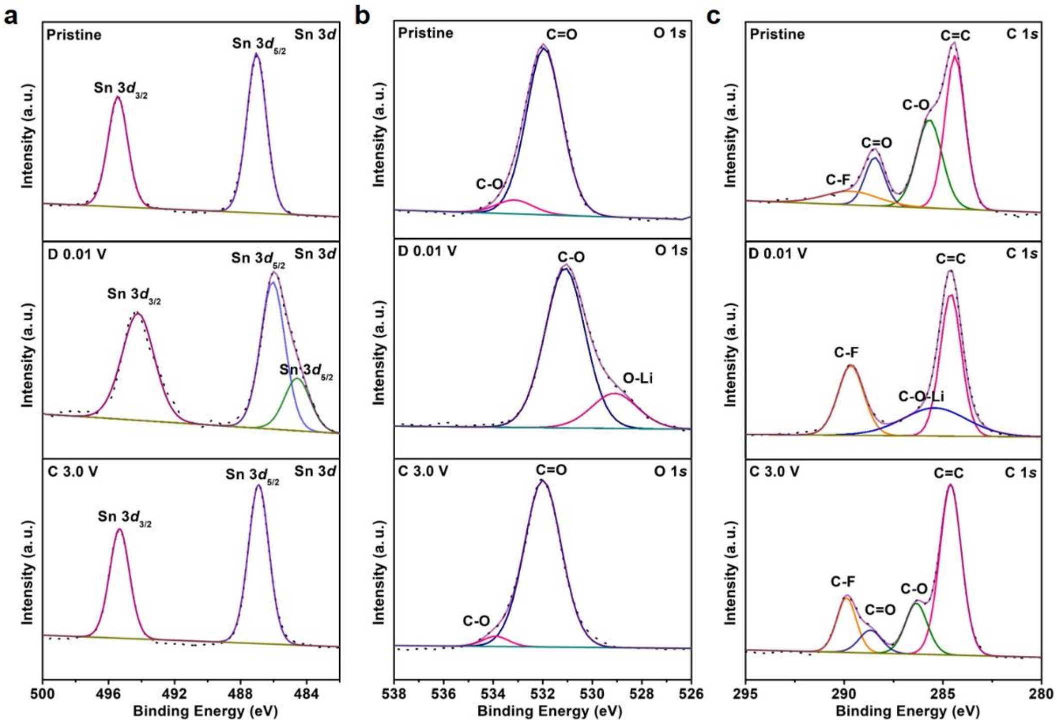

Supplementary Figure 17. Ex situ Sn 3*d* (a), O 1*s* (b) and C 1*s* (c) XPS spectra of Sn_2(dobdc) electrode at selected states.

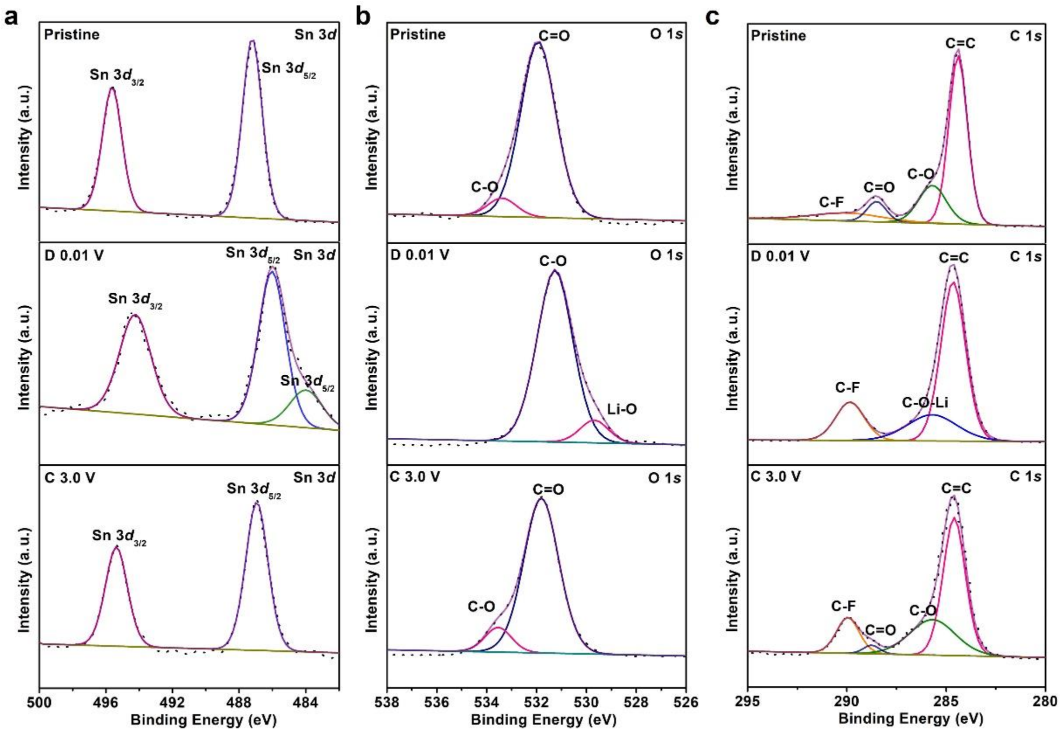

Supplementary Figure 18. Ex situ Sn 3*d* (a), O 1*s* (b) and C 1*s* (c) XPS spectra of Sn_2(dobpdc) electrode at selected states.

Supplementary Notes

After etching treatment to remove the SEI layer, the ex situ Sn 3*d*, O 1*s* and C 1*s* XPS spectra of Sn_2(dobdc) and Sn_2(dobpdc) in pristine, fully discharged, and fully charged states were measured (Supplementary Figures 17 and 18). For Sn 3*d* spectra of Sn_2(dobdc) (Supplementary Figure 17a), compared with the peaks at 495.4 and 487.0 eV for Sn^{2+} in the pristine state, the peaks moved to 494.2 and 486.0 eV corresponding to Sn^0 in the discharged electrode[S5]. Furthermore, the weak signal at 484.6 eV assigned to Sn-Li constitution emerged in the discharged electrode, in accordance with the alloying reaction of Sn with Li ions[S6]. When being recharged to 3.0 V, the peaks at 495.1 and 486.8 eV dominated, demonstrating the recovery of Sn^{2+}. For the O 1*s* spectra (Supplementary Figure 17b), the peaks at 532.0 and 533.1 eV assigned to the C=O bond of carboxylate group and the C–O bond of phenolate group in pristine state[S7,S8]. After full discharge, the C=O bond disappeared, whereas the peak at 531.1 eV for C–O bond and a new peak for Li–O bond at 529.1 eV were observed[S9], suggesting that carboxylate group and phenolate group on MOF ligands participated in the lithium storage reaction and thus the C–O–Li bonding formed. When recharged to 3.0 V, the peak at 532.0 eV for C=O bond and the peak at 533.7 eV for C–O bond recovered because of the extraction of Li ions from carboxylate group and phenolate group. In addition, the analyses of C 1*s* spectra at different states were conducted to further investigate the redox reactions of carboxylate group and phenolate group with Li ions (Supplementary Figure 17c). In the pristine state, the peaks of C=O and C–O bonds were located at 288.5 and 285.7 eV. After full discharge, the peak of C=O at disappeared and the peak at 285.4 eV corresponding to the C–O–Li was observed[S8,S10]. When recharged to 3.0 V, the peaks of C=O (288.7 eV) and C–O (286.1 eV) recovered. Therefore, both O 1*s* and C 1*s* XPS spectra indicated the reversible evolution of carboxylate group and phenolate group for lithium storage reaction.

The changes in the XPS spectra for the Sn_2(dobpdc) electrode are similar to those for the Sn_2(dobdc) electrode (Supplementary Figure 18).

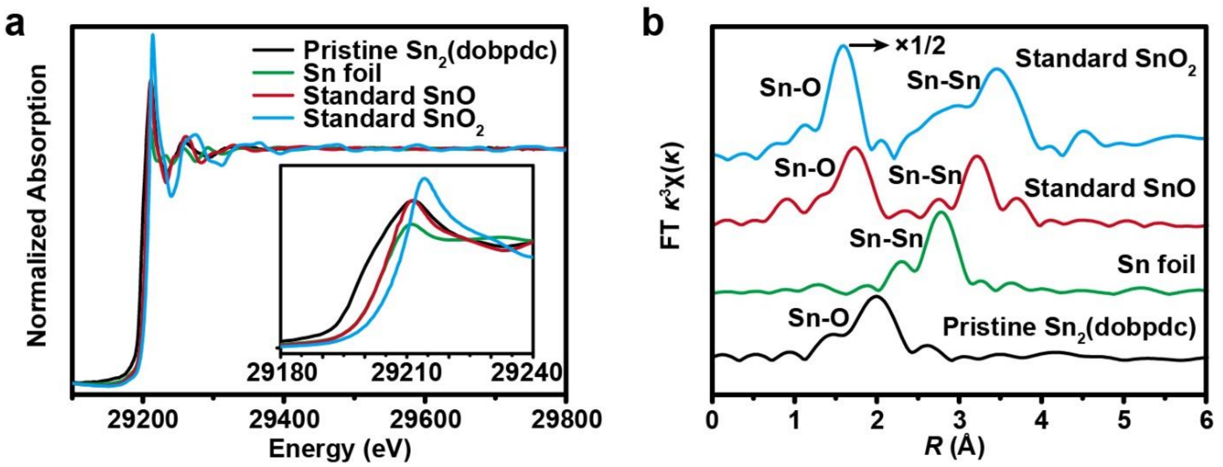

Supplementary Figure 19. XAFS results. (a) Synchrotron Sn K-edge XANES spectra of pristine Sn_2(dobpdc), Sn foil, standard SnO and SnO_2. Inset: enlarged spectra at Sn K-edge. (b) Sn K-edge k^3-weighted FT-EXAFS spectra of pristine Sn_2(dobpdc), Sn metal, SnO and SnO_2 (the EXAFS intensity of SnO_2 is shown at half value).

As shown in Supplementary Figure 19a, the XANES spectra of Sn foil, standard SnO and SnO_2 are much different from that of the pristine Sn_2(dobpdc), probably because of the formation of SnO_4 coordination environment in the Sn_2(dobpdc). However, the Sn^{2+} state in pristine Sn_2(dobpdc) can be clearly identified because the intensity of white line in Sn_2(dobpdc) is very close to that of main peak in SnO. The Fourier transformations (FT) of k^3-weighted for EXAFS oscillations for the radial structure functions of pristine Sn_2(dobpdc), Sn foil, standard SnO and SnO_2 are demonstrated in Supplementary Figure 19b. The pristine Sn_2(dobpdc) exhibits the characteristic Sn-O coordination with intensive peak at around 2.13 Å, which coincides well with the lengths of Sn-O bonds obtained from the single crystal X-ray diffraction data (Supplementary Table 3). The absence of Sn-Sn bonds suggests that there were no metal Sn in pristine Sn_2(dobpdc) sample.

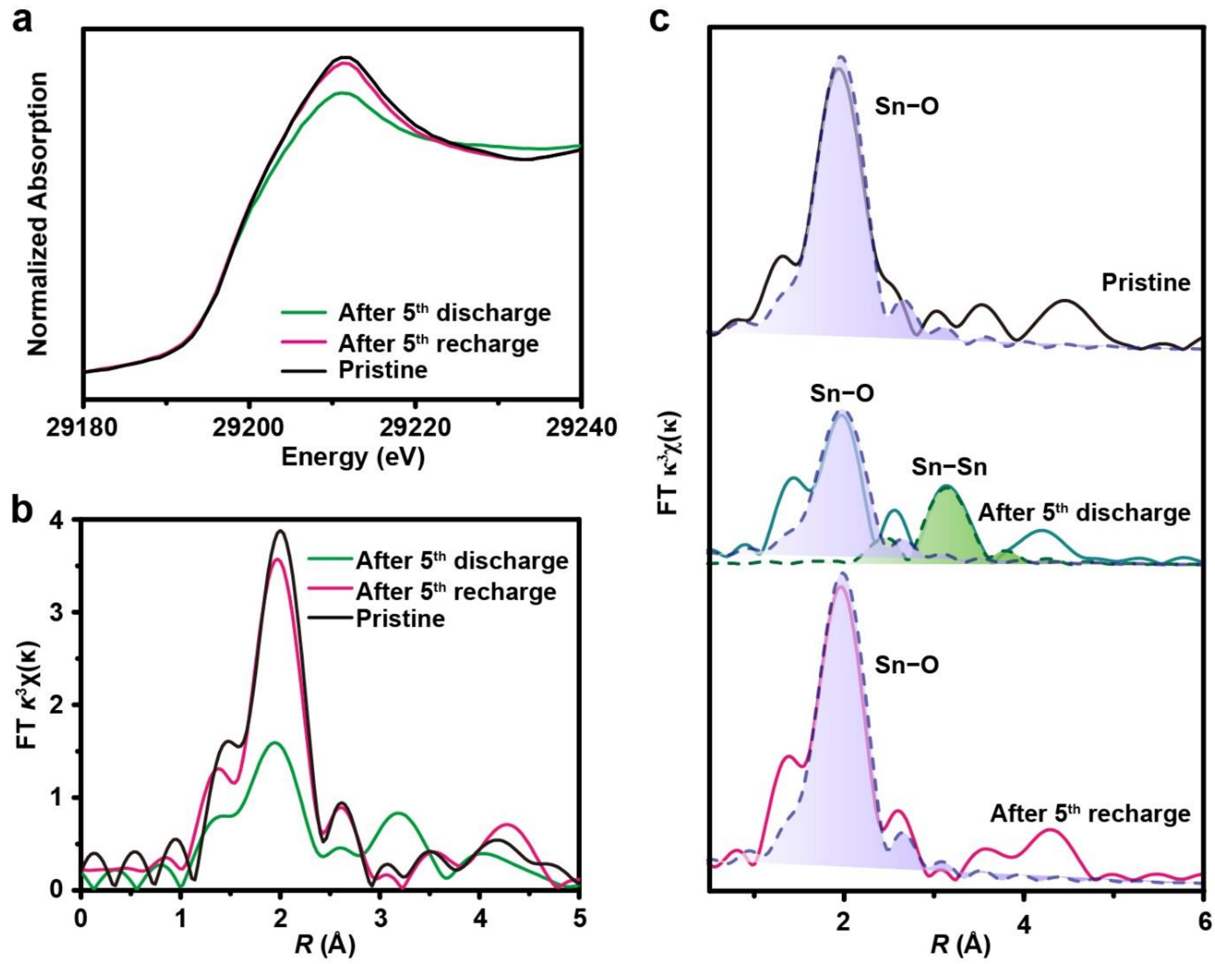

Supplementary Figure 20. (a) Synchrotron Sn K-edge XANES spectra of pristine Sn_2(dobpdc), after the fifth fully discharged, and after the fifth fully charged. (b) Sn K-edge k^3-weighted FT-EXAFS spectra of pristine Sn_2(dobpdc), after the fifth fully discharged, and after the fifth fully charged. (c) Corresponding EXAFS fitting results of (b).

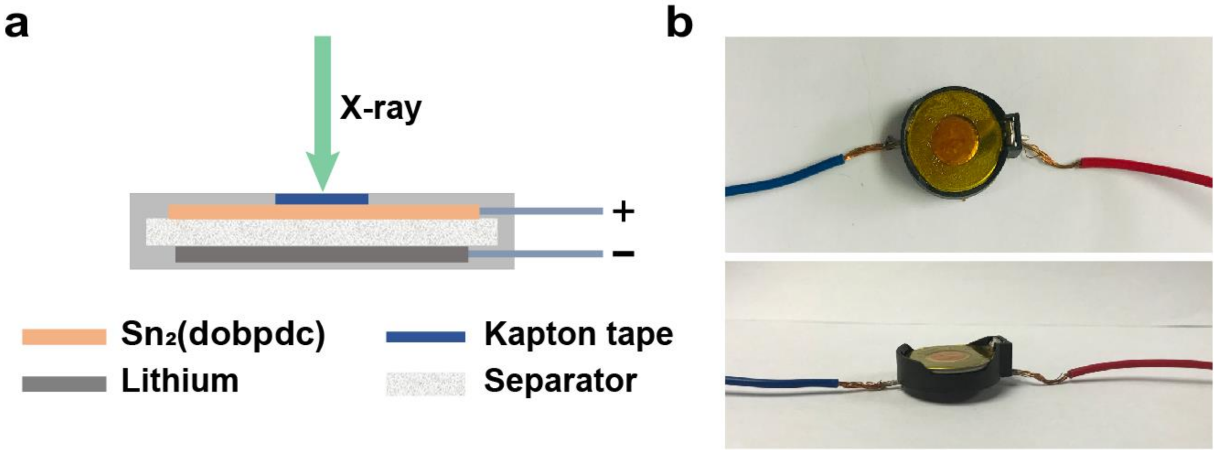

Supplementary Figure 21. (a) Schematic cross-section of a coin-type battery for the quasi-in situ XAFS test. (b) The real cell pictures for quasi-in situ XAFS test.

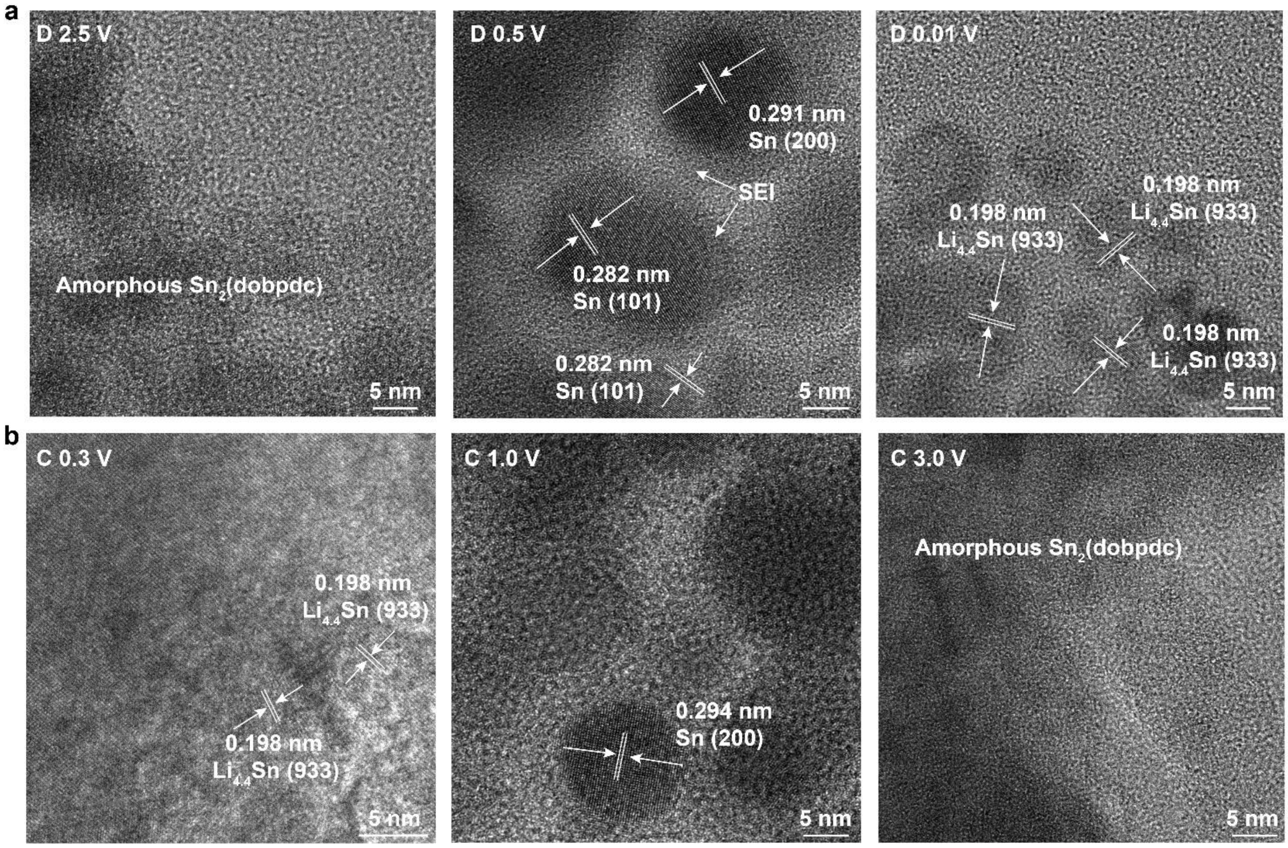

Supplementary Figure 22. Ex situ HRTEM images at different states during the second lithiation-delithiation cycle of Sn_2(dobpdc). (**a**) HRTEM images of Sn_2(dobpdc) in a pristine state, discharged to 0.7 V and discharged to 0.01 V. (**b**) HRTEM images of Sn_2(dobpdc) after being charged to 0.3 V, charged to 1.0 V and charged to 3.0 V.

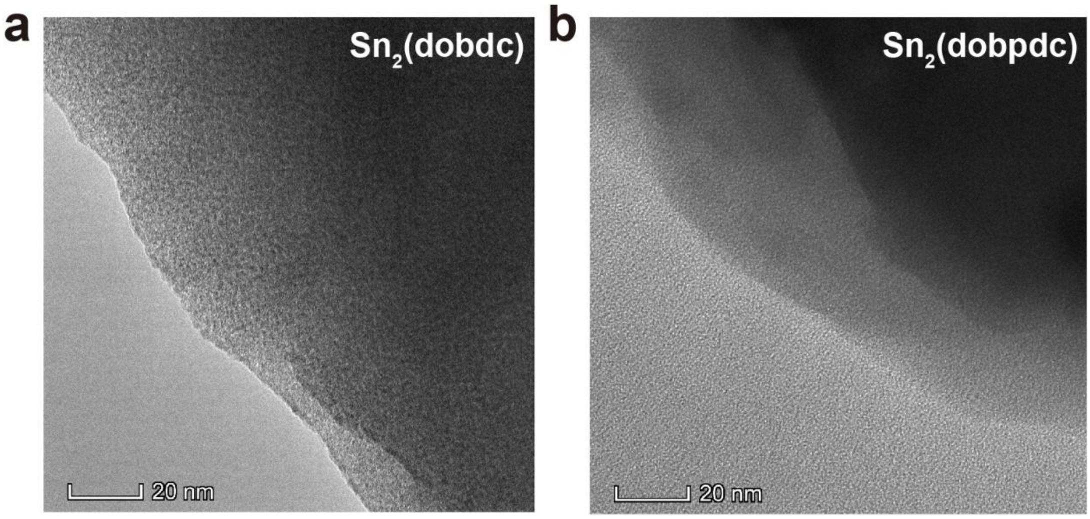

Supplementary Figure 23. HRTEM images of pristine Sn_2(dobdc) (a) and Sn_2(dobpdc) (b) powders.

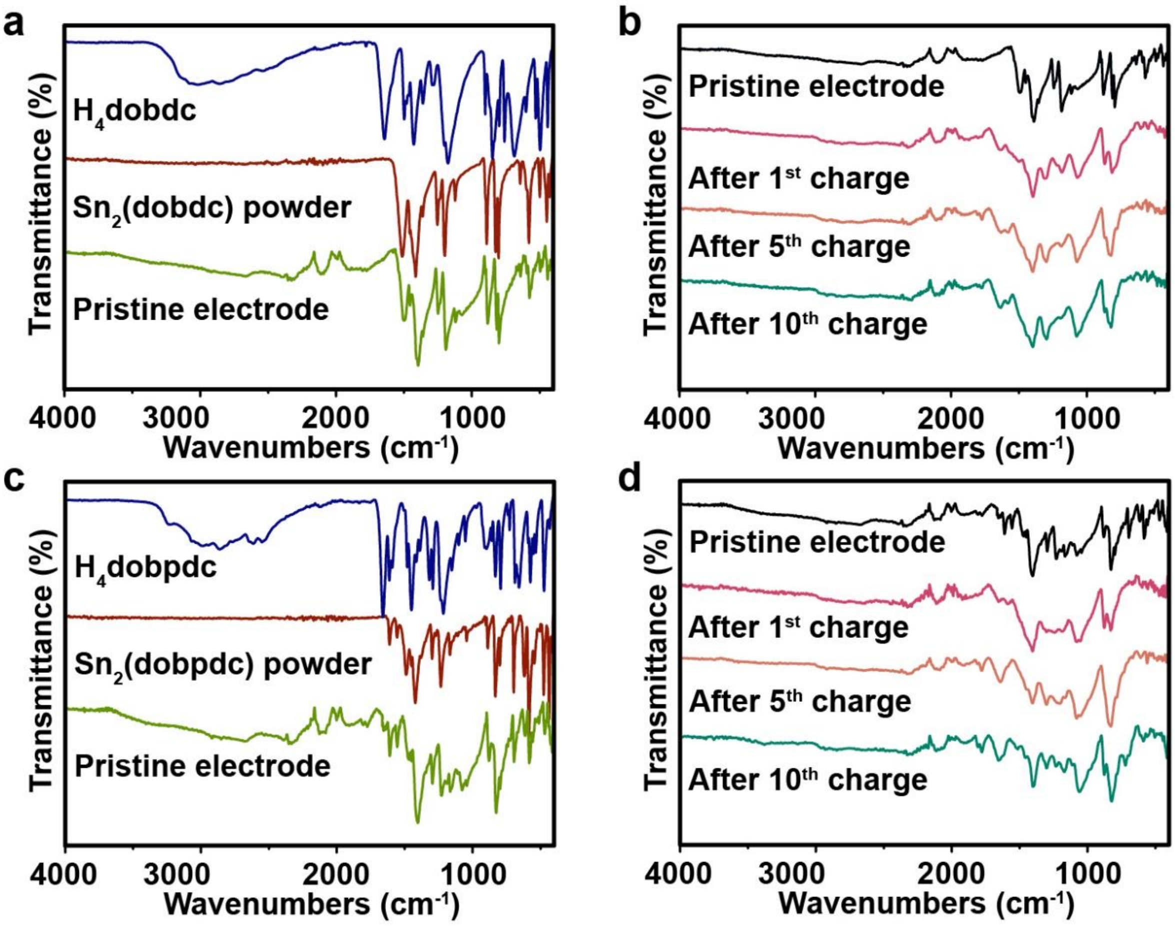

Supplementary Figure 24. FTIR spectra. Ex situ FTIR spectra of Sn_2(dobdc) at different states before (a) and after cycling (b). Ex situ FTIR spectra of Sn_2(dobpdc) before (c) and after cycling (d).

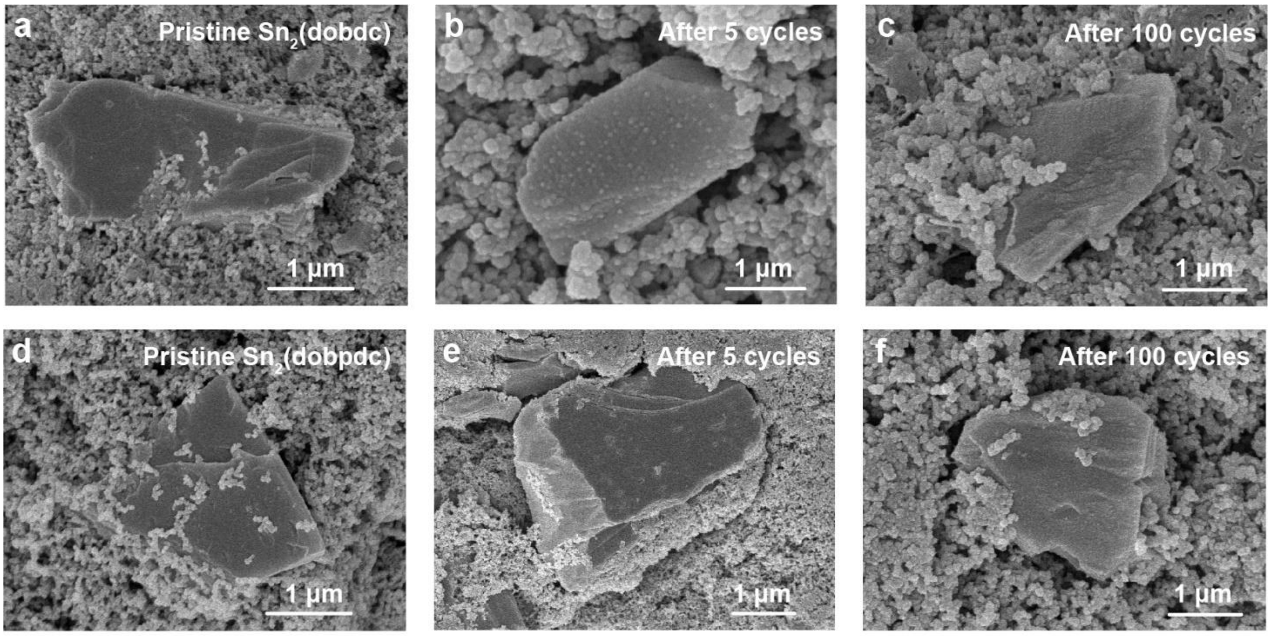

Supplementary Figure 25. Morphology characterizations. SEM images of Sn_2(dobdc) (a-c) and Sn_2(dobpdc) (d-f) collected at different states.

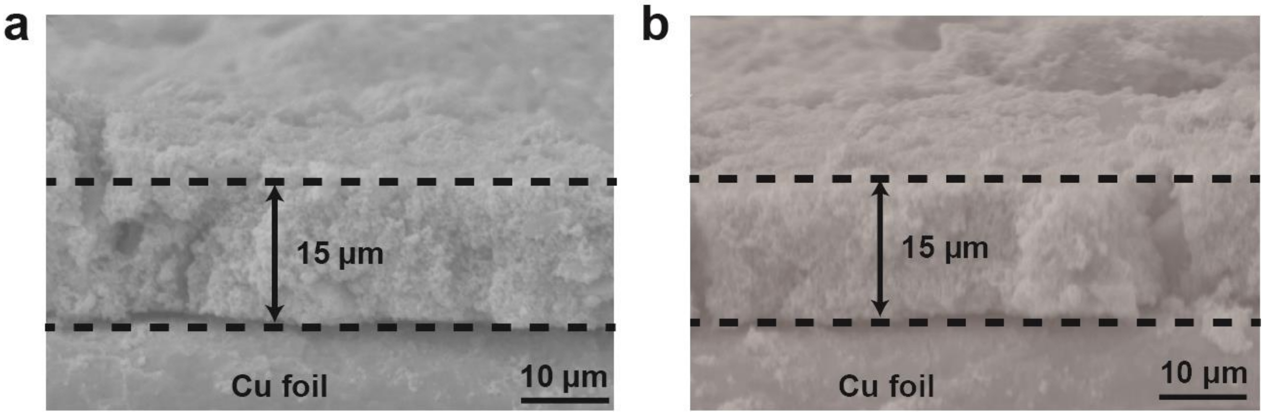

Supplementary Figure 26. Electrode thickness of Sn_2(dobdc) (a) and Sn_2(dobpdc) (b) electrodes.

Supplementary Table 1. Comparison of recent MOFs as anodes for LIBs.

MOFs	RC ($mAh\ g^{-1}$) / CR ($mA\ g^{-1}$)	Cycle number	Refs.
$Sn_2(dobdc)$	731 / 200	200	This work
$Sn_2(dopbdc)$	1018 / 200	200	This work
Mn-UMOFNs	1187 / 100	100	S11
Mn-BDC	974 / 100	100	S12
CoBTC-EtOH	856 / 100	100	S13
Mn-BTC	694 / 100	100	S14
NENU-507	640 / 100	100	S15
$[Co_3(L^1)(N_3)_4]$	580 / 100	200	S15
$[Co_4L^2(N_3)(H_2O)_2]$	595 / 100	200	S16
$[Mn_4L^2(N_3)_6(H_2O)_2]$	595 / 100	200	S16
Ni-UMOFNs	546 / 100	100	S11
$[Mn_2(L^1)(N_3)_2(H_2O)_2]\cdot 3H_2O$	358 / 100	200	S16
$[Cd(HTCPPA)\cdot 2H_2O]_n$	302 / 100	100	S17
$Cu_3(BTC)_2$	474 / 383	50	S18
$Co_2(OH)_2BDC$	650 / 50	100	S19
Ni-MOF	620 / 100	100	S20
Mn(3,5-PDC) $2H_2O$	554 / 100	240	S21
Mn(2,5-FDC) $3H_2O$	436 / 100	206	S21
$[Co(C_8H_4O_4)_4]_n$	700 / 60	100	S22
$Zn_3(HCOO)_6$	560 / 60	60	S23
$Co_3(HCOO)_6$	410 / 60	60	S23
NNU-11	750 / 50	200	S24
Co-LCP	545 / 50	50	S25
Mn-LCP	390 / 50	50	S26
$Ni-Me_4bpz$	120 / 50	100	S27

RC: Reversible capacity. CR: Current Rate.

Supplementary Table 2. Crystal data and structural refinement parameters for Sn_2(dobdc) and Sn_2(dobpdc).

	**[Sn_2(dobdc)]$_n$**	**[Sn_2(dobpdc)]$_n$**
Formula	C_4HO_3Sn	$C_7H_3O_3Sn$
Fw	215.76	253.78
Temp, K	120.00(10)	120.00(10)
Crystal syst	Monoclinic	Monoclinic
Space group	$P2_1/c$	$P2_1$/c
a, Å	7.4141(3)	10.8951(3)
b, Å	7.5679(2)	7.5313(2)
c, Å	9.1100(3)	8.8733(3)
α, deg	90	90
ß, deg	106.603	92.329(3)
γ, deg	90	90
V, $Å^3$	489.84	727.49(4)
Z	4	4
*D*c, g/cm^3	2.925	2.317
μ, mm^{-1}	5.107	3.458
Data/ parameter	857 / 73	1277 / 100
obs reflns	2814	4619
θ range, °	7.126 - 50.002	6.578 - 49.992
R_{int}/GOF on F^2	0.0418 / 1.021	0.0468 / 1.063
R_1, wR_2[I>2σ (I)]	0.0232, 0.0407	0.0253, 0.0417
R_1, wR_2(all data)	0.0329, 0.0447	0.0340, 0.0463
max/min, e $Å^{-3}$	0.58 / -0.68	0.64 / -0.65

Supplementary Table 3. Selected bond lengths (Å) and angles (º) for Sn_2(dobdc) and Sn_2(dobpdc).

**Sn_2(dobdc)**			
Sn1-O3^1	2.195(3)	O3^1-Sn1-O1	80.18(12)
Sn1-O2	2.183(3)	O3^1-Sn1-O1^2	78.00(12)
Sn1-O1^2	2.382(3)	O2-Sn1-O3^1	85.59(13)
Sn1-O1	2.222(3)	O2-Sn1-O1^2	72.67(12)
		O2-Sn1-O1	77.00(12)
		O1-Sn1-O1^2	143.65(7)

Symmetry transformations used to generate equivalent atoms: ^{1}+X, 3/2-Y, -1/2+Z; ^{2}1-X, 1/2+Y, 1/2-Z; ^{3}+X, 3/2-Y, 1/2+Z; ^{4}1-X, -1/2+Y, 1/2-Z; ^{5}2-X, 1-Y, 1-Z.

**Sn_2(dobpdc)**			
Sn1-O3^1	2.181(3)	O3^1-Sn1-O1	81.59(11)
Sn1-O2	2.116(8)	O3^1-Sn1-O1^2	79.55(10)
Sn1-O1^2	2.104(6)	O2-Sn1-O3^1	83.28(10)
Sn1-O1	2.065(6)	O2-Sn1-O1^2	71.45(9)
		O2-Sn1-O1	77.56(10)
		O1-Sn1-O1^2	145.23(6)

Symmetry transformations used to generate equivalent atoms: ^{1}+X, 3/2-Y, -1/2+Z; ^{2}1-X, 1/2+Y, 1/2-Z; ^{3}+X, 3/2-Y, 1/2+Z; ^{4}1-X, -1/2+Y, 1/2-Z; ^{5}-X,1-Y,1-Z.

Supplementary Table 4. Cartesian coordinates of Li_8dobdc and Li_{12}dobpdc for DFT calculation.

Cartesian coordinates for Li_8dobdc			
Element	Coordinates		
	X	Y	Z
O	3.726424	0.642739	-0.275760
O	3.221470	-1.498581	-0.535850
O	1.553859	2.322339	0.518391
C	2.801745	-0.279485	-0.226161
C	0.572344	-1.289576	0.365570
H	0.986574	-2.141050	0.917040
C	0.854192	1.262153	0.343834
C	1.450358	-0.072227	0.132073
O	-3.726232	-0.642573	-0.275118
O	-3.221079	1.498458	-0.536083
O	-1.553319	-2.322564	0.516312
C	-2.801689	0.279541	-0.225703
C	-0.571766	1.290659	0.363537
H	-0.986094	2.142178	0.914961
C	-0.853179	-1.262169	0.342710
C	-1.449865	0.072574	0.132276
Li	-0.012742	-0.000714	2.057254
Li	0.001254	0.000401	-1.458559
Li	1.979344	-2.798545	-0.592796
Li	4.939017	-0.725681	-0.724666
Li	3.318525	2.345750	0.273871
Li	-3.317545	-2.346947	0.270346
Li	-4.938512	0.726246	-0.726475
Li	-1.976776	2.796661	-0.597628

Cartesian coordinates for Li_{12}dobpdc							
Element	Coordinates			Element	Coordinates		
	X	Y	Z		X	Y	Z
O	-4.03291	-2.31359	0.23475	H	0.13111	-2.38594	0.0588
O	-5.55591	-0.76408	-0.2938	C	0.70888	-0.27231	-0.01423
O	-4.64352	1.96555	0.17109	Li	-5.16576	3.83152	0.20041
C	-4.32359	-1.10101	0.00652	Li	-6.83905	-1.8848	-1.166
C	-3.25277	-0.07899	0.04194	Li	-4.25966	-3.99602	0.89521
C	-3.45999	1.38327	0.07717	Li	4.25864	3.99389	-0.90035
C	-2.29117	2.18649	0.03403	Li	6.83421	1.89492	1.17198
H	-2.40942	3.26436	0.05628	Li	5.16276	-3.83246	-0.22732
C	-1.8479	-0.56388	0.08046	Li	-6.16543	0.87377	0.28646
H	-1.7434	-1.63788	0.10716	Li	-1.97473	0.94335	1.93406
C	-0.93925	1.6708	-0.02928	Li	6.169	-0.87739	-0.25511
H	-0.13137	2.3859	-0.06896	Li	1.9815	-0.93278	-1.93685
C	-0.70876	0.27233	0.01154	Li	-2.17049	0.90793	-1.86423
O	4.03437	2.31286	-0.2354	Li	2.16796	-0.92024	1.86355
O	5.55426	0.76479	0.30524				
O	4.64368	-1.96578	-0.17212				
C	4.32378	1.10082	-0.00262				
C	3.25285	0.07874	-0.04077				
C	3.46008	-1.38331	-0.07897				
C	2.29104	-2.18655	-0.04033				
H	2.40921	-3.26437	-0.06519				
C	1.84816	0.56395	-0.07986				
H	1.74371	1.63803	-0.10351				
C	0.93919	-1.67086	0.02306				

Supplementary Table 5. Calculated capacities and utilization efficiencies of Sn_2(dobdc) and Sn_2(dobpdc).

MOFs	Sn_2(dobdc)	Sn_2(dobpdc)
Theoretical number of Li uptake in organic ligand	8	12
Theoretical number of Li uptake in metal center	8.8	8.8
Theoretical Capacity (mAh g^{-1})	1044	1099
Reversible Capacity at 200 mA g^{-1} (mAh g^{-1})	731	1018
Utilization Efficiency (%)	70.0	92.6
Number of accessible active sites	11.7	19.3

Supplementary Table 6. Sn K-edge EXAFS fitting results (*R*: distance; CN: coordination number; σ^2: Debye-Waller factor; ΔE_0: inner potential correction; D: discharge; C: charge) for Sn_2(dobpdc) electrode.

Sample	Sn-O		Sn-Sn		σ^2 (Å^2)	ΔE_0 (eV)
	R (Å)	CN	*R* (Å)	CN		
Pristine	2.13±0.02	3.7±0.7	—	—	0.01(Sn-O)<br>0.015(Sn-Sn)	11±1(Sn-O)<br>3±1(Sn-Sn)
D 1.5 V	2.14±0.02	2.8±0.5	—	—		
D 0.01 V	—	—	2.92±0.03	6.3±1.2		
C 0.6 V	—	—	2.93±0.03	2.5±0.6		
C 3.0 V	2.14±0.02	2.7±0.4	—	—		
5th full discharge	2.15±0.02	1.8±0.3	2.95±0.02	3.1±0.8		
5th full charge	2.15±0.02	3.5±0.6	—	—		

Supplementary Notes

For the explanation of unrecovered coordination number of Sn–O shell at the 1st cycle: Firstly, the incomplete recovery of Sn–O shell at the 1st cycle is due to the formation of SEI layer. At the 1st cycle, the SEI layer formed on the Sn-MOF surfaces by the side reaction of electrolyte with lithium ions[S28], which has the features of high lithium-ion conductivity and negligible electronic conductivity[S29]. The electronically insulating property of the SEI layer prevents sustained reduction of the electrolyte on the electrode surface, while the ion conductive nature allows permeation of lithium ions to the Sn-MOF and provides pathways for the desired ion transport [S30]. The formation SEI layer might prohibit the reformation of a small segment of Sn–O bonds. Secondly, the phenomenon of incomplete recovery of Sn–O shell at the 1st cycle has also been observed in other literatures[S31,S32], which was attributed to the amorphization of the active material during after lithiation and delithiation process.

Supplementary Table 7. Selected Sn-based anodes and other typical anode materials for LIBs

Active materials	Structure or type	Preparation method	RC / CR	Cycle number	Refs.
Sn_2(dobdc)	Sn atoms anchored on organic ligands	One-step hydrothermal method	731 / 200	200	This work
Sn_2(dopbdc)	Sn atoms anchored on organic ligands	One-step hydrothermal method	1018 / 200	200	This work
3D Sn scaffold	Sn hollow spheres	Multistep templating	885 / 200	200	S33
TiO_2-Sn@CNFs	Nano-Sn dispersed in carbon nanofibers and TiO_2 pipes	Electrospinning and atomic layer deposition	831 / 100	200	S34
Co–Sn intermetallic electrodes	Highly ordered mesoporous Co_xSn_y framework	Nanoreplication method	530 / 50	50	S35
SnO_2-Fe-G nanocomposites	SnO_2-Fe aggregates binding on graphite nanosheets	Two-step ball milling	1338 / 200	400	S36
Sn@C nanoboxes	Sn nanoparticles encapsulated in hollow carbon nanobox	Sacrificial template method	810 / 200	500	S37
Polydopamine -coated SnO_2	SnO_2 nanocrystals coated with polydopamine	Sacrificial template method	921 /200	300	S38
SnO_2@MOF/graphene composite.	SnO_2 nanoparticles packed into Al-MOF wrapped by graphene	Wet impregnation method and assembly process	450 / 1000	1000	S39
Porous hard carbons	Insertion type	Thermal annealing method	310 / 5000	10000	S40
Fe_2O_3 microboxes	Conversion type	Thermal annealing method	950 / 200	30	S41
Co_3O_4	Conversion type	Hybrid assembly and chemical reduction approaches	1000 / 74	130	S42

RC: Reversible capacity (mAh g^{-1}). CR: Current Rate (mA g^{-1})

Supplementary Methods:

1. Analysis of capacitive contribution

For the analysis of Li^+ storage mechanism in **Supplementary Figure 11**:

The peak currents (i) and scan rates (v) have the following relationship[S43,S44]:

$$i = av^b, \tag{1}$$

which can be rewritten as

$$\log(i) = b\log(v) + \log(a) \tag{2}$$

where a and b are constants. When b value is close to 0.5, the electrochemical process is controlled by Li^+ diffusion. While the b value reaches to 1, the capacitive effects will dominate the charge/discharge process. By fitting the plots of log(i) vs. log(v) (Supplementary Figures 11c and 11d), the calculated b values of peak 1, 2, and 3 for Sn_2(dobdc) are 0.62, 0.62, and 0.63, respectively. Correspondingly, the battery with Sn_2(dobpdc) showed the calculated b values of peak 1, 2, and 3 are 0.91, 0.63, and 0.65. The ratios of Li^+ capacitive contribution can be further quantitatively calculated through the following equation:

$$i = k_1v + k_2v^{1/2} \tag{3}$$

where i is the current response, k_1v is the capacitive effects contribution, and $k_2v^{1/2}$ is the diffusion-controlled contribution. By determining both k_1 and k_2 constants, the fraction of the current from capacitive effect and diffusion behavior contribution can be distinguished (Supplementary Figures 11e and 11f).

2. Analysis of GITT results

The Li^+ diffusion coefficients (D_{Li+}) in Sn-MOFs electrodes are calculated by GITT method according to the equation[S45-47]:

$$D = \frac{4}{\pi\tau}\left(\frac{m_B V_M}{M_B S}\right)^2\left(\frac{\Delta E_s}{\Delta E_t}\right)^2 \tag{4}$$

where τ, m_B, V_M, S, and M_B are the pulse time (s), the mass of Sn-MOFs in the electrode (g), molar volume of Sn-MOFs (cm^3 mol^{-1}), the contact area between electrode and electrolyte, and the molecular weight of Sn-MOFs (g mol^{-1}), respectively. ΔE_s is the voltage difference between the steady state and the initial state of every step and ΔE_τ is the change of total voltage during a pulse step excluding the IR drop. The molar volumes of Sn_2(dobdc) and Sn_2(dobpdc) can be obtained from the crystal data in Supplementary Table 2.

3. Electrical conductivity measurement

The electrical conductivities of Sn-MOFs were acquired by measuring the pressed pellets using CHI 660E electrochemical workstation[S45]. The pressed pellets were obtained under 12 MPa for 10 min (Supplementary Figure 7). Then each pellet was tightly sandwiched between two stainless-steel gaskets, and the resistance value (R) was obtained by the linear sweep voltammetry (LSV) measurement in the voltage range from 0–0.1 V. The electrical conductivities (σ) of the Sn-MOFs pellets were calculated using the equation: $\sigma = 1/\rho = L/(R \times S)$, where ρ is the resistivity of pellet, L and S are the pellet thickness and area. As shown in Supplementary Figure 8, the electrical conductivities of Sn_2(dobdc) and Sn_2(dobpdc) are 2.9×10^{-7} and 9.8×10^{-7} S cm^{-1}, respectively.

Supplementary References

S1 Lei, Z. et al. Boosting lithium storage in covalent organic framework via activation of 14-electron redox chemistry. *Nat. Commun.* **9**, 576 (2018).

S2 Darago, L. E., Aubrey, M. L., Yu, C. J., Gonzalez, M. I. & Long, J. R. Electronic conductivity, ferrimagnetic ordering, and reductive insertion mediated by organic mixed-valence in a ferric semiquinoid metal-organic framework. *J. Am. Chem. Soc.* **137**, 15703–15711 (2015).

S3 Qian, J. et al. High rate and stable cycling of lithium metal anode. *Nat. Commun.* **6**, 6362 (2015).

S4 Chen, L., Wang, K., Xie, X. & Xie, J. Effect of vinylene carbonate (VC) as electrolyte additive on electrochemical performance of Si film anode for lithium ion batteries. *J. Power Sources* **174**, 538–543 (2007).

S5 Kang, S., Chen, X. & Niu, J. Sn wears super skin: a new design for long cycling batteries. *Nano Lett.* **18**, 467–474 (2018).

S6 Jiang, C. et al. A multi-ion strategy towards rechargeable sodium-ion full batteries with high working voltage and rate capability. *Angew. Chem. Int. Ed.* **57**, 16370–16374 (2018).

S7 Wang, L. et al. A two-dimensional metal-organic polymer enabled by robust nickel-nitrogen and hydrogen bonds for exceptional sodium-ion storage. *Angew. Chem. Int. Ed.* **59**, 22126–22131 (2020).

S8 Sun, T. et al. A biodegradable polydopamine-derived electrode material for high-capacity and long-life lithium-ion and sodium-ion batteries. *Angew. Chem. Int. Ed.* **55**, 10662–10666 (2016).

S9 Jiang, L. et al. Inhibiting solvent co-intercalation in a graphite anode by a localized high-concentration electrolyte in fast-charging batteries. *Angew. Chem. Int. Ed.* **60**, 3402–3406 (2021).

S10 Wang, Y., Qu, Q. Liu, G., Battaglia, V. S. & Zheng, H. Aluminum fumarate-based metal organic frameworks with tremella-like structure as ultrafast and stable anode for lithium-ion batteries. *Nano Energy* **39**, 200–210 (2017).

S11 Li, C. et al. Ultrathin manganese-based metal-organic framework nanosheets: low-cost and energy-dense lithium storage anodes with the coexistence of metal and ligand redox activities. *ACS Appl. Mater. Interfaces* **9**, 29829–29838 (2017).

S12 Hu, H. P. et al. A thermally activated manganese 1,4-benzenedicarboxylate metal organic framework with high anodic capability for Li-ion batteries. *New J. Chem.* **40**, 9746–9752 (2016).

S13 Li, C. et al. High anodic performance of Co 1, 3, 5-benzenetricarboxylate coordination polymers for Li-ion battery. *ACS Appl. Mater. Interfaces* **8**, 15352–15360 (2016).

S14 Maiti, S., Pramanik, A., Manju U. & Mahanty, S. Reversible lithium storage in manganese 1,3,5-benzenetricarboxylate metal-organic framework with high capacity and rate performance. *ACS Appl. Mater. Interfaces* **7**, 16357–16363 (2016).

S15 Wang, Y. Y. et al. Diamondoid-structured polymolybdate-based metal–organic frameworks as high-capacity anodes for lithium-ion batteries. *Chem. Commun.* **53**, 5204–5207 (2017).

S16 Gong, T., Lou, X., Gao, E. & Hu, B. Pillared-layer metal–organic frameworks for improved lithium-ion storage performance. *ACS Appl. Mater. Interfaces* **9**, 21839–21847 (2017).

S17 Lin, X. M. et al. Lithium-ion-battery anode materials with improved capacity from a metal-organic framework. *Inorg. Chem.* **55**, 8244–8247 (2016).

S18 Maiti, S., Pramanik, A., Manju, U. & Mahanty, S. Cu_3(1,3,5-benzenetricarboxylate)$_2$ metal-organic framework: a promising anode material for lithium-ion battery. *Microporous Mesoporous Mater.* **226**, 353–359 (2016).

S19 Gou, L. et al. One-pot synthesis of a metal–organic framework as an anode for Li-ion batteries with improved capacity and cycling stability. *J. Solid State Chem.* **210**, 121–124 (2014).

S20 Zhang, Y. et al. A nickel-based metal-organic framework: a novel optimized anode material for Li-ion batteries. *Mater. Lett.* **161**, 712–715 (2015).

S21 Fei, H. L., Liu, X., Li, Z. W. & Feng, W. J. Metal dicarboxylates: new anode materials for lithium-ion batteries with good cycling performance. *Dalton Trans.* **44**, 9909–9914 (2015).

S22 Wang, L. P. et al. Metal organic framework derived cobalt dicarboxylate as a high capacity anode material for lithium-ion batteries. *Energy Technol.* **5**, 634–642 (2017).

S23 Saravanan, K., Nagarathinam, M., Balaya P. & Vittal, J. J. Lithium storage in a metal organic framework with diamondoid topology–a case study on metal formats. *J. Mater. Chem.* **20**, 8329–8335 (2010).

S24 Huang, Q. et al. A highly stable polyoxometalate-based metal-organic framework with π-π stacking for enhancing lithium ion battery performance. *J. Mater. Chem. A* **5**, 8477–8483 (2017).

S25 Shi, C. D. et al. Synthesis of cobalt-based layered coordination polymer nanosheets and their application in lithium-ion batteries as anode materials. *RSC Adv*. **6**, 4442–4447 (2016).

S26 Liu, Q. et al. Manganese-based layered coordination polymer: synthesis, structural characterization, magnetic property, and electrochemical performance in lithium-ion batteries. *Inorg. Chem.* **52**, 2817–2822 (2013).

S27 An, T. et al. A flexible ligand-based wavy layered metal–organic framework for lithium-ion storage. *J. Colloid Interf. Sci.* **445**, 320–325 (2015).

S28 Qian, J. et al. High rate and stable cycling of lithium metal anode. *Nat. Commun.* **6**, 6362 (2015).

S29 Jurng, S., Brown, Z. L., Kim, J. & Luchi, B. L. Effect of electrolyte on the nanostructure of the solid electrolyte interphase (SEI) and performance of lithium metal anodes. *Energy Environ. Sci.* **11**, 2600–2608 (2018).

S30 Shi, S. et al. Direct calculation of Li-ion transport in the solid electrolyte interphase. *J. Am. Chem. Soc.* **134**, 15476–15487 (2012).

S31 Wang, L. et al. Novel preparation of N-doped SnO_2 nanoparticles via laser-assisted pyrolysis: demonstration of exceptional lithium storage properties. *Adv. Mater.* **29**, 1603286 (2016).

S32 Kim, H. et al. New insight into the reaction mechanism for exceptional capacity of ordered mesoporous SnO_2 electrodes via synchrotron-based X-ray analysis. *Chem. Mater.* **26**, 6361–6370 (2014).

S33 Liu, J. et al. High volumetric capacity three-dimensionally sphere-caged secondary battery anodes. *Nano Lett.* **16**, 4501–4507 (2016).

S34 Mao, M. et al. Pipe-wire TiO_2-Sn@carbon nanofibers paper anodes for lithium and sodium ion batteries. *Nano Lett.* **17**, 3830–3836 (2017).

S35 Park, G. O. et al. Discovering a dual-buffer effect for lithium storage: durable nanostructured ordered mesoporous Co-Sn intermetallic electrodes. *Adv. Funct. Mater.* **26**, 2800–2808 (2016).

S36 Hu, R. et al. Stabilizing the nanostructure of SnO_2 anodes by transition metals: a route to achieve high initial coulombic efficiency and stable capacities for lithium storage. *Adv. Mater.* **29**, 1605006 (2017).

S37 Zhang, H. et al. Tailored yolk-shell Sn@C nanoboxes for high-performance lithium storage. *Adv. Funct. Mater.* **27**, 1606023 (2017).

S38 Jiang, B. et al. Polymer-templated formation of polydopamine-coated SnO_2 nanocrystals: anodes for cyclable lithium-ion batteries. *Angew. Chem. Int. Ed.* **56**, 1869–1872 (2017).

S39 Gao, C. et al. Optimized assembling of MOF/SnO_2/Graphene leads to superior anode for lithium ion batteries. *Nano Energy* **74**, 104868 (2020).

S40 Lv, W. et al. Peanut shell derived hard carbon as ultralong cycling anodes for lithium and sodium batteries. *Electrochim. Acta* **176**, 533–541 (2015).

S41 Zhang, L. et al. Formation of Fe_2O_3 microboxes with hierarchical shell structures from metal-organic frameworks and their lithium storage properties. *J. Am. Chem. Soc.* **134**, 17388–17391 (2012).

S42 Yang, S., Feng, X., Ivanovici, S. & Mullen, K. Fabrication of graphene-encapsulated oxide nanoparticles: towards high-performance anode materials for lithium storage. *Angew. Chem. Int. Ed.* **49**, 8408–8411 (2010).

S43 Chao, D. et al. Array of nanosheets render ultrafast and high-capacity Na-ion storage by tunable pseudocapacitance. *Nat. Commun.* **7**, 12122 (2016).

S44 Wan, F. et al. Aqueous rechargeable zinc/sodium vanadate batteries with enhanced performance from simultaneous insertion of dual carriers. *Nat. Commun.* **9**, 1656 (2018).

S45 Shi, R. et al. Nitrogen-rich covalent organic frameworks with multiple carbonyls for high-performance sodium batteries. *Nat. Commun.* **11**, 178 (2020).

S46 Weppner, W. & Huggins, R. A. Determination of the kinetic parameters of mixed-conducting electrodes and application to the system Li_3Sb. *J. Electrochem. Soc.* **124**, 1569–1578 (1977).

S47 Yang, X. & Rogach, A. L. Electrochemical techniques in battery research: a tutorial for nonelectrochemists. *Adv. Energy Mater.* **9**, 1900747 (2019).
